# Anticancer and Anti-Neuroinflammatory Constituents Isolated from the Roots of *Wasabia japonica*

**DOI:** 10.3390/antiox11030482

**Published:** 2022-02-28

**Authors:** Jong Eel Park, Tae Hyun Lee, Song Lim Ham, Lalita Subedi, Seong Min Hong, Sun Yeou Kim, Sang Un Choi, Chung Sub Kim, Kang Ro Lee

**Affiliations:** 1 School of Pharmacy, Sungkyunkwan University, Suwon 16419, Korea; jongilpark@skku.edu (J.E.P.); thlee16@skku.edu (T.H.L.); 2 Korea Environment Corporation, 42 Hwangyeong-ro, Seo-gu, Incheon 22689, Korea; 3 Department of Biopharmaceutical Convergence, Sungkyunkwan University, Suwon 16419, Korea; songlim0824@skku.edu; 4 Gachon Institute of Pharmaceutical Science, Gachon University, Incheon 21936, Korea; subedilali@gmail.com (L.S.); hongsm0517@gmail.com (S.M.H.); sunnykim@gachon.ac.kr (S.Y.K.); 5 College of Pharmacy, Gachon University, #191, Hambakmoero, Yeonsu-gu, Incheon 21936, Korea; 6 Korea Research Institute of Chemical Technology, Daejeon 34114, Korea; suchoi@krict.re.kr

**Keywords:** *Wasabia japonica*, Brassicaceae, 2-butenolide, anticancer, anti-neuroinflammation

## Abstract

Wasabi (*Wasabia japonica* (Miq.) Matsum.) is a pungent spice commonly consumed with sushi and sashimi. From the roots of this plant, a new 2-butenolide derivative (**1**) and 17 previously reported compounds (**2**–**18**) were isolated and structurally characterized. Their chemical structures were characterized based on the conventional NMR (^1^H and ^13^C, COSY, HSQC, and HMBC) and HRESIMS data analysis. All of these phytochemicals (**1**–**18**) were evaluated for their antiproliferative effects on the four human tumor cell lines (A549, SK-OV-3, SK-MEL-2, and MKN-1), for their inhibitory activity on nitric oxide (NO) production in lipopolysaccharide (LPS)-activated BV-2 microglia cells, and for their nerve growth factor (NGF)-releasing effect from C6 glioma cells. Among the isolated compounds, compound **15** showed powerful antiproliferative activities against A549 and SK-MEL-2 cell lines with IC_50_ values of 2.10 and 9.08 μM, respectively. Moreover, the new compound **1** exhibited moderate NO inhibition activity with IC_50_ value of 45.3 μM.

## 1. Introduction

*Wasabia japonica* (Miq.) Matsum., commonly known as wasabi, is one of the most well-known species among Brassicaceae plants. This plant is a perennial plant and has been cultivated mainly in Korea and Japan. Because of their tangy taste, the roots of wasabi have long been used as a spice for sushi and sashimi. A wide range of biological studies on *W. japonica* have been investigated so far; however, most of the experiments were focused on its sulfur-containing constituents, isothiocyanates (ITCs). For examples, two major components, 6-(methylsulfinyl)hexyl isothiocyanate (6-MITC) and allyl isothiocyanate (AITC), exhibited anticancer [1,2,3,4,5], antioxidant [6], anti-inflammatory [7,8], neuroprotective [9], and antimicrobial [10] properties. The other minor constituents of *W. japonica* contain antioxidant phenylpropanoid [11], anticancer and anti-inflammatory monogalactosyl diacylglycerides [12], and antifungal indole derivatives [13]. However, minor bioactive components of *W. japonica* with different structural classes other than ITCs remain largely unknown.

**Figure 1:**
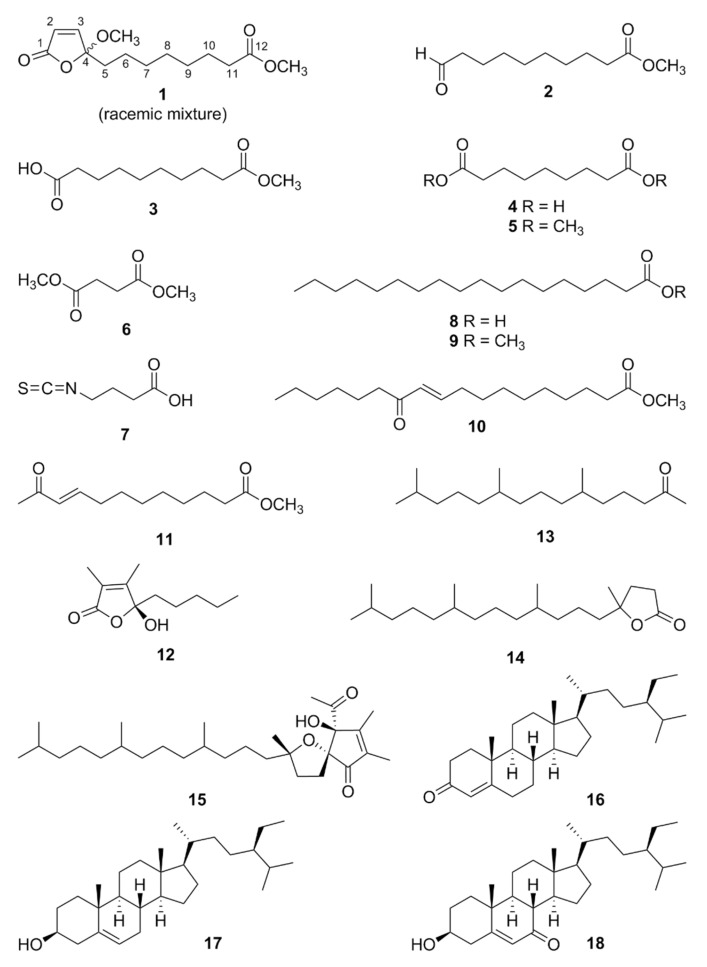
Chemical structure of compounds **1**–**18**.

In our continuing efforts to discover bioactive constituents from the Korean traditional medicinal plants, we have investigated the roots of *W. japonica* and isolated structurally unique thioglycosides and lignan glycosides with neurotrophic and/or anti-inflammatory activities [14,15]. To discover anticancer compounds within non-ITCs class from this plant, we have tested antiproliferative effects of hexanes-, chloroform (CHCl_3_)-, ethyl acetate (EtOAc)-, and *n*-butanol (*n*-BuOH)-soluble fractions of the methanol (MeOH) extract of *W. japonica* roots, and among them, the hexanes-soluble faction displayed the most potent activities with GI_50_ values of 30.57, 21.71, 16.34, and 50.64 μg/mL against A549, SK-OV-3, SK-MEL-2, and HCT-15 cancer cell lines, respectively (Table 1). Therefore, we then focused mainly on the hexanes-soluble fraction, and herein we report the isolation and structure characterization of anticancer and anti-inflammatory constituents from the *W. japonica* roots. A total 18 compounds were identified (Figure 1), and the structure of the new isolate, wasabolide (**1**), was elucidated by the conventional spectroscopic (i.e., NMR) and spectrometric (i.e., MS) data analysis.

## 2. Materials and Methods

### 2.1. General Experimental Procedures

Bruker AVANCE III 700 NMR spectrometer at 700 MHz (^1^H) and 175 MHz (^13^C) was used to measure the NMR spectra (^1^H, ^13^C, COSY, HSQC, and HMBC) at 700 MHz (^1^H) and 175 MHz (^13^C) with chemical shifts given in ppm (δ) (Bruker, Karlsruhe, Germany) and the resultant spectra were processed using MestReNova (Mnova, version 14.1.2-25024). Chloroform-*d* and methanol-*d*_4_ (Cambridge Isotope Laboratory, Inc., Tewksbury, MA, USA) were used for NMR analysis of the isolated compounds. High-resolution electrospray ionization mass spectroscopy (HRESIMS) was measured by using an Agilent 1290 Infinity II HPLC instrument (Foster City, CA, USA) coupled to a G6545B quadrupole time-of-flight (Q-TOF) mass spectrometer (Agilent Technologies, Foster City, CA, USA) with a Kinetex C_18_ 5 µm column (250 mm length × 4.6 mm i.d.; Phenomenex, Torrance, CA, USA). The semipreparative high-performance liquid chromatography (HPLC) furnished with a Gilson 306 pump (Gilson, Middleton, WI, USA), a Luna C18 10 μm column (250 mm length × 10 mm i.d.; Phenomenex, Torrance, CA, USA), an Apollo Silica 5 μm column (250 mm length × 10 mm i.d.; Apollo, Manchester, UK), and a Shodex refractive index detector (Gilson, New York, NY, USA) was used for compounds isolation. Low pressure liquid chromatography (LPLC) was performed with a LiChroprep Lobar-A Si 60 column (Merck, Darmstadt, Germany) and an FMI QSY-0 pump. Column chromatography was performed employing either silica gel 60 (70−230 and 230−400 mesh; Merck, Darmstadt, Germany) or RP-C_18_ silica gel (Merck, 230−400 mesh). Merck precoated silica gel F_254_ plates and RP-C_18_ F_254s_ plates (Merck, Darmstadt, Germany) were used for thin-layer chromatography (TLC) analysis. Spots were detected on TLC under UV light or by heating after spraying with anisaldehyde−sulfuric acid.

### 2.2. Plant Material

The roots of *W. japonica* (3.3 kg) were collected in Hanam, Republic of Korea, in October 2014. One of the authors, Kang Ro Lee, identified the plant, and a voucher specimen (SKKU-NPL 1409) was deposited in the herbarium of the School of Pharmacy, Sungkyunkwan University, Suwon, Republic of Korea.

### 2.3. Extraction and Isolation

The roots of *W. japonica* (3.3 kg) were extracted with 80% aqueous MeOH at room temperature and filtered. The crude extract was concentrated under reduced pressure to yield a MeOH extract (750 g). The extract was suspended in deionized H_2_O, and then it was partitioned with hexanes (1.3 g), CHCl_3_ (5.6 g), EtOAc (5.8 g), and *n*-BuOH (2.7 g). The hexanes-soluble fraction (1.3 g) was chromatographed on silica gel column (hexane-EtOAc, 4:1 → 1:1) to afford 20 fractions (H1-H20). Fraction H1 (100 mg) was applied to Waters Sep-pak silica Vac 6 cc (hexane-EtOAc, 1:0 → 1:1) to yield six sub-fractions (H11–H16), and compound **9** (2 mg, 0.0003%) was acquired by purifying sub-fraction H15 (10 mg), employing semipreparative silica HPLC with an isocratic mixture of hexane-EtOAc (30:1, flow rate of 2.0 mL/min). Compound **13** (3 mg, 0.0004%) was purified from the fraction H2 (35 mg), using semipreparative silica HPLC eluting with hexane-EtOAc (30:1, 2 mL/min) under isocratic conditions. Fraction H3 (35 mg) was purified by using semipreparative silica HPLC, with an isocratic mixture of hexane-EtOAc (15:1, 2 mL/min), to produce compounds **10** (3 mg, 0.0004%) and **15** (3 mg, 0.0004%). Fraction H4 (33 mg) was divided into four sub-fractions (H41–H44), using C_18_ Waters Sep-pak Vac 6 cc by eluting with 95% aqueous MeOH. Compound **16** (3 mg, 0.0004%) was isolated from sub-fraction H44 (15 mg), using semipreparative silica HPLC eluting with an isocratic mixture of hexane-EtOAc (30:1, 2 mL/min). Fraction H5 (63 mg) was subjected to C_18_ Waters Sep-pak Vac 6 cc with 95% aqueous MeOH as an eluent to acquire three sub-fractions (H51–H53). Compounds **2** (4 mg, 0.0005%) and **5** (2 mg, 0.0003%) were purified from sub-fraction H51 (40 mg). Compound **14** (2 mg, 0.0003%) was isolated by purification, using semipreparative silica HPLC eluting with an isocratic mixture of hexane–EtOAc (8:1, 2 mL/min) from sub-fraction H52 (11 mg). Fraction H6 (100 mg) was separated on a Lobar A Si 60 (240 mm × 10 mm) column (CHCl_3_-MeOH, 500:1) to yield four sub-fractions (H61–H64). Sub-fraction H64 (19 mg) was further separated by using semipreparative silica HPLC eluting with an isocratic mixture of hexane–EtOAc (4:1, 2 mL/min) to acquire compound **17** (8 mg, 0.001%). Compound **11** (2 mg, 0.0003%) was purified from fraction H7 (13 mg) by employing semipreparative HPLC (85% aqueous MeOH, 2 mL/min) under isocratic conditions. Fraction H9 (40 mg) was separated into two sub-fractions (H91 and H92) through C_18_ Waters sep-pak Vac 6 cc by eluting with 100% MeOH, and sub-fraction H91 (15 mg) was further isolated by semipreparative HPLC (82% aqueous MeOH, 2 mL/min) under isocratic conditions to obtain compound **12** (2 mg, 0.0003%). Fraction H10 (40 mg) was applied to C_18_ Waters Sep-pak Vac 6 cc with 100% MeOH as an eluent to furnish three sub-fractions (H101–H103). Sub-fraction H101 (21 mg) was purified by semipreparative silica HPLC eluting with an isocratic mixture of hexane-EtOAc (2.5:1, 2 mL/min) to obtain compound **1** (3 mg, 0.0004%). Fraction H11 (28 mg) was isolated into three sub-fractions (H111–H113) using C_18_ Waters Sep-pak Vac 6 cc by eluting with 100% MeOH. Among the sub-fractions, compound **8** (4 mg, 0.0005%) was purified from the sub-fraction H112 through semipreparative silica HPLC eluting with an isocratic mixture of hexane-EtOAc (2.5:1, 2 mL/min). Fraction H13 (45 mg) was divided to three sub-fractions (H131–H133) by C_18_ Waters Sep-pak Vac 6 cc by eluting with 100% MeOH and compound **18** (5 mg, 0.0007%) was obtained by purification of sub-fraction H133 (16 mg), using semipreparative silica HPLC eluting with an isocratic mixture of hexane-EtOAc (1:1, 2 mL/min). The EtOAc-soluble fraction (5.8 g) was fractionated into six fractions (E1-E6) by passage over Diaion HP-20 column with step gradient solvents of MeOH-H_2_O (0:1, 1:4, 2:3, 3:2, 4:1, and 1:0) as eluents. Fraction E2 (300 mg) was applied to a Lobar-A RP-18 (240 mm × 10 mm) column (20% aquous MeOH) to produce four sub-fractions (E21–E24). Sub-fraction E22 (80 mg) was further isolated into seven sub-fractions (E211–E217) through a Lobar A Si 60 (240 mm × 10 mm) column (CHCl_3_-MeOH-H_2_O, 3:1:0.15). Compound **7** (3 mg, 0.0004%) was isolated from sub-fraction E222 (15 mg) by semipreparative silica HPLC eluting with an isocratic mixture of CHCl_3_-MeOH-H_2_O (5:1:0.1, 2 mL/min). Sub-fraction E24 (30 mg) was purified by using semipreparative HPLC (10% aqueous CH_3_CN, 2 mL/min) under isocratic conditions to afford compound **6** (2 mg, 0.0003%). Fraction E5 (160 mg) was chromatographed on silica gel column chromatography (EtOAc-MeOH-H_2_O, 3:1:0.15 → 1:1:0.1) to furnish five sub-fractions (E51–E55). Compound **3** (4 mg, 0.0005%) was isolated by purification of sub-fraction E51 (10 mg), using semipreparative HPLC (50% aqueous MeOH, 2 mL/min) under isocratic conditions. Through the purification of sub-fraction E52 (17 mg) by using semipreparative HPLC (45% aqueous MeOH, 2 mL/min) under isocratic conditions, compound **4** (3 mg, 0.0004%) was obtained.

*Wasabolide* (**1**): colorless gum; ^1^H (700 MHz) and ^13^C (175 MHz) NMR data in chloroform-*d* (see Table 2); HRESIMS (positive-ion mode) *m/z* 271.1535 [M + H]^+^ (calcd. for C_14_H_23_O_5_, 271.1540, error = 1.8 ppm), 293.1348 [M + Na]^+^ (calcd. for C_14_H_22_NaO_5_, 293.1365, error = 3.8 ppm).

### 2.4. Sulforhodamine B Colorimetric Assay

The antiproliferative activities of isolated compounds (**1**–**18**) were tested against the A549 (non-small-cell lung adenocarcinoma), SK-OV-3 (ovary malignant ascites), SK-MEL-2 (skin melanoma), HCT-15 (human colon adenocarcinoma), and MKN-1 (adenosquamous carcinoma), utilizing the SRB method as previously reported (Table 3) [17]. Etoposide (≥98%; Sigma-Aldrich, St. Lous, MO, USA) served as a positive control.

### 2.5. Nitric Oxide (NO) Assay

The nitric oxide (NO) assay was performed analogously, as described in Reference [18]. The BV-2 cells, which were developed by V. Bocchini at the University of Perugia (Perugia, Italy), were used for this study [19,20]. The cells were seeded in a 96-well plate (4 × 10^4^ cells/well) and incubated in the presence or absence of various doses of tested compounds (**1**–**18**). Lipopolysaccharide (LPS) (100 ng/mL) was added to all the pretreated wells, except the control one, and grown for 1 day. The produced levels of nitrite (NO_2_), a soluble oxidized product of NO, was evaluated with 0.1% *N*-1-napthylethylenediamine dihydrochloride and 1% sulfanilamide in 5% phosphoric acid, aka the Griess reagent. The supernatant (50 μL) was mixed with the Griess reagent (50 μL). After 10 min, the absorbance was gauged at 570 nm. *N*^G^-monomethyl-l-arginine (l-NMMA), as a nitric oxide synthase (NOS) inhibitor, was used as a positive control. Graded sodium nitrite solution was utilized to determine nitrite concentrations. A 3-[4,5-dimethylthiazol-2-yl]-2,5-diphenyltetrazolium bromide (MTT) assay was used for the cell-viability assay (Table 4).

### 2.6. Nerve Growth Factor (NGF) Assay

The nerve growth factor (NGF) assay was performed analogously, as described in Reference [21]. C6 glioma cell lines were used to measure the NGF of the culture medium containing 10% fetal bovine serum (FBS) and 1% penicillin–streptomycin (PS) in 5% CO_2_ incubator. The cells were seeded in a 24-well culture plate (1 × 10^5^ cells/well) and incubated for 24 h. The cells were treated with or without 20 µM of the compounds (**1**–**18**), together with serum-free Dulbecco’s modified Eagle’s medium (DMEM) for another 24 h. Released NGF levels from the supernatants from each cell were measured by using an ELISA development kit (R&D System, Minneapolis, MN, USA). Moreover, the cell viability was evaluated by using MTT assay; 6-shogaol used as a positive control, and the results are expressed as percentage of the control group.

## 3. Results and Discussion

### 3.1. Structure Elucidation of Compounds ***1**–**18***

**Figure 2:**
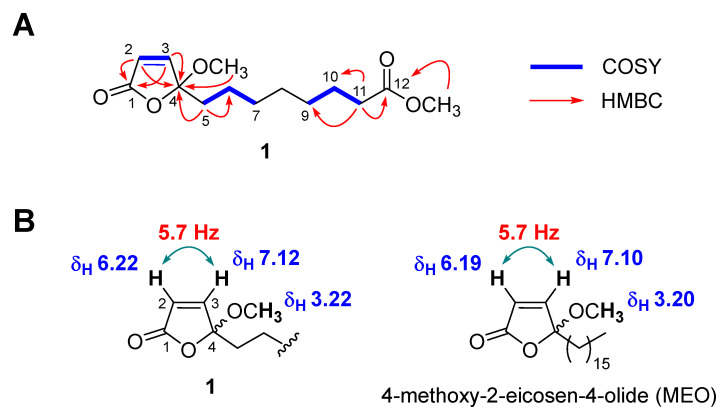
Structure elucidation of **1**. (**A**) Key COSY and HMBC correlation of **1**. (**B**) Comparison of the key ^1^H NMR data around the UBL functionality in **1** (left) and its structurally similar marine metabolite MEO (right).

Wasabolide (**1**) was obtained as a colorless gum, and its molecular formula was established as C_14_H_22_O_5_ from the protonated and sodiated molecular ions at *m/z* 271.1535 [M + H]^+^ (calcd. for C_14_H_23_O_5_^+^, 271.1540, error = 1.8 ppm) and 293.1348 [M + Na]^+^ (calcd. for C_14_H_22_NaO_5_^+^, 293.1359, error = 3.8 ppm), respectively, in the HRESIMS data (Figure S1). The ^1^H NMR spectrum of **1** (Figure S2) suggested the presence of two olefinic [δ_H_ 7.12 (1H, d, *J* = 5.7 Hz, H-3) and 6.22 (1H, d, *J* = 5.7 Hz, H-2)], two methoxy [δ_H_ 3.66 (3H, s, OCH_3_-12) and 3.22 (3H, s, OCH_3_-4)], and seven methylene [δ_H_ 2.29 (2H, t, *J* = 7.5 Hz, H-11), 1.89 (2H, m, H-5), 1.61 (2H, m, H-10), 1.37 (2H, m, H-6), and 1.30 (6H, overlap, H-7–H-9)] protons in **1**. The ^13^C NMR spectrum of **1** (Figure S3) showed total 14 resonances, which was consistent with HRESIMS data mentioned above, including two ester/carboxylic acid(s) (δ_C_ 174.4 (C-12) and 170.1 (C-1)), two double bond [δ_C_ 153.7 (C-3) and 125.0 (C-2)], a deoxygenated [δ_C_ 111.4 (C-4)], two methoxy [δ_C_ 51.6 (OCH_3_-12) and 51.3 (OCH_3_-4)], and seven methylene [δ_C_ 37.1 (C-5), 34.2 (C-11), 29.4 (C-7), 29.13 (C-9), 29.07 (C-8), 25.0 (C-10), and 23.3 (C-6)] carbon peaks. To elucidate the planar structure of **1**, 2D NMR data of **1** including COSY, HSQC, and HMBC (Figures S4–S6) were obtained and analyzed. The presence of an α,β-unsaturated γ-butyrolactone (UBL, = 2-butenolide) moiety in **1** was suggested by observing COSY correlation between H-2 and H-3 and HMBC cross-peaks between H-2/H-3 and C-1 and H-2/H-3/H-5 and C-4 (Figure 2A). The HMBC correlation between OCH_3_-4 and C-4 indicated a methoxy group located at the γ-position of UBL moiety (Figure 2A). In addition, an unusually smaller coupling constant between the *cis*-oriented two olefinic protons H-2 and H-3, 5.7 Hz, was observed. The similar *J* value has been reported from other UBL-containing organic molecules [16,22,23], supporting the presence of UBL functionality in **1**. The remaining COSY correlations between H-6 and H-5/H-7 and H-10 and H-9/H-11 and HMBC cross-peaks between H-5 and C-6, H-11 and C-9/C-10/C-12, and OCH_3_-12 and C-12 indicated the presence of a fully saturated aliphatic chain with a terminal methyl ester group as the other substructure of **1**. Therefore, the planar structure of **1** was determined as in Figure 1. The most structurally similar molecule to **1** was found to be 4-methoxy-2-eicosen-4-olide (MEO) isolated from a marine sponge (Figure 2B, right) [16] by the literature search, using SciFinder. MEO shared most of the NMR features, with **1** especially at the UBL functionality (Table 2 and Figure 2B), thus supporting our structural assignment of **1**. Finally, it was concluded that **1** was a racemic mixture based on the almost zero value of specific rotation and no Cotton effect observation in electronic circular dichroism (ECD) spectrum of **1**.

The 17 known compounds **2**–**18** were identified as methyl 10-oxodecanoate (**2**) [24], monomethyl sebacate (**3**) [25], azelaic acid (**4**) [26],dimethyl azelate (**5**) [26], dimethyl succinate (**6**) [27], 4-isothiocyanatobutanoic acid (**7**) [28], octadecanoic acid (**8**) [29], methyl stearate (**9**) [30], (10*E*)-12-oxo-10-octadecenoic acid methyl ester (**10**) [31], methyl 11-oxo-9-dodecenoate (**11**) [32], (*S*)-5-hydroxy-3,4-dimethyl-5-pentylfuran-2(5*H*)-one (**12**) [33], 6,10,14-Trimethyl-2-pentadecanone (**13**) [34], 5-methyl-5-(4,8,12-trimethyl-tridecyl)-dihydro-furan-2-one (**14**) [35], α-tocospiro A (**15**) [36], stigmast-4-en-3-one (**16**) [37], β-sitosterol (**17**) [38], and 7-oxo-β-sitosterol (**18**) [39] by comparing their spectroscopic data (Figures S7–S24) with those in the literature.

### 3.2. Biosynthetic Proposal of the New Compound ***1***

**Figure 3:**
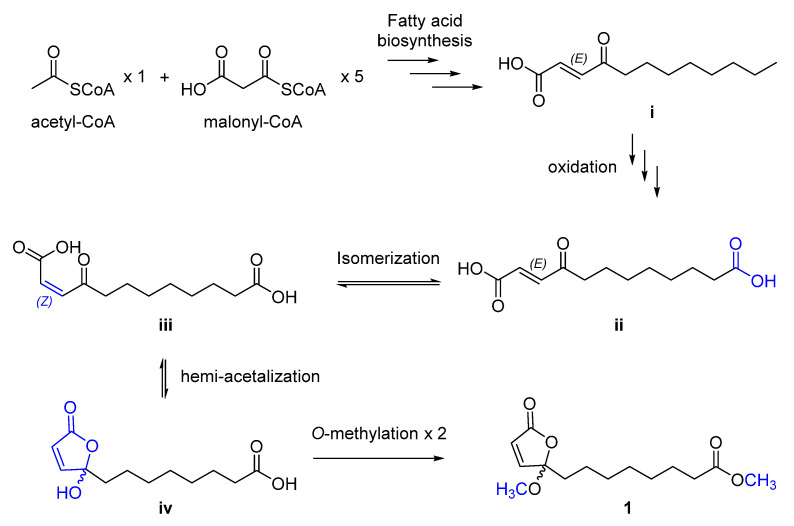
Plausible biosynthetic pathway of **1**.

Upon the structural characterization of the new metabolite **1**, its biosynthetic pathway was proposed as described below (Figure 3). First, one unit of acetyl-CoA and five units of malonyl-CoA could form the C12-fatty acid **i** with a double bond and a carbonyl group at α, β- and γ-position, respectively, which reaction resembled typical fatty acid biosynthesis. Then, oxidation of the terminal methyl group in **i** to carboxylic acid could afford dicarboxylic acid **ii** [40,41]. Geometrical isomerization could occur in **ii** that transforms the *E*- to *Z*-configuration of the double bond to yield **iii**. A hemi-acetalization reaction on **iii** could produce **iv** [22,42], which contains UBL moiety, and, finally, **1** could be formed by *O*-methylation at both hydroxy and carboxylic groups in **iv**.

### 3.3. Antiproliferative Activity of the Isolated Compounds *(**1**–**18**)*

In line with the potent antiproliferative activity of the hexanes-soluble fraction mentioned above, the isolated compounds (**1**–**18**) were evaluated for their antiproliferative activity against four human tumor cell lines, namely A549 (non-small cell lung adenocarcinoma), SK-OV-3 (ovary malignant ascites), SK-MEL-2 (skin melanoma), and MKN-1 (adenosquamous carcinoma), by SRB assay. Compounds **10**, **11**, **14**–**16**, and **18** isolated from the hexanes-soluble fraction strongly inhibited proliferation in cancer cell lines with different selectivity (Table 3). In detail, α-tocospiro A (**15**) showed potent activity against A549 cell line with IC_50_ value of 2.10 μM, which was comparable to that of the positive control substance, etoposide (1.51 μM). While the chemical structures of **13**–**15** are similar in that these three molecules had the same C15 fully saturated farnesyl unit, we observed no and weaker activity of **13** (IC_50_ > 20 μM) and **14** (IC_50_ = 13.28 μM), respectively, on the same cancer cell line. These data suggested that the bulky spirobicyclic moiety in α-tocospiro A (**15**) would play an important role in exhibiting antiproliferative activities in A549 cell line. Interestingly, Yuan et al. reported that α-tocospiro A (**15**) had no inhibitory activity on the A549 proliferation (IC_50_ > 20 μM) [43], but we observed the significant potency of α-tocospiro A (**15**) in this study. Moreover, α-tocospiro A (**15**) also displayed a strong antiproliferative effect on SK-OV-2, SK-MEL-2, and MKN-1 cell lines, with IC_50_ values ranging from 9.08 to 13.23 μM. Compounds **10**, **11**, **14**, **16**, and **18** showed antiproliferative activities on several cancer cell lines with IC_50_ values between 12.86 to 26.72 μM. The other compounds were inactive (IC_50_ > 30 μM).

### 3.4. Anti-Neuroinflammatory Activity of the Isolated Compounds *(**1**–**18**)*

The potential anti-neuroinflammatory effects of the isolates (**1**−**18**) were tested by measuring the level of NO production in LPS-stimulated BV-2 microglia cells. As shown in Table 4, among the tested phytochemicals, the new compound **1** showed the strongest inhibitory activity on the NO production, with an IC_50_ value of 45.3 μM and without significant cell toxicity up to 20 μM. Compounds **5**, **11**, and **15** also exhibited weak activity with IC_50_ value of 90.0, 59.6, and 92.4 μM, respectively. The other compounds were inactive (IC_50_ > 100 μM).

### 3.5. Neurotrophic Activity of the Isolated Compounds *(**1**–**18**)*

Lastly, the neurotrophic effect of compounds **1–18** was also evaluated, and compound **17** displayed a weak effect on NGF release from C6 cells, with a stimulation level of 107.51 ± 5.38%, compared to the positive control substance, 6-shogaol (149.53 ± 5.38%). The other compounds were inactive (stimulation level < 100%).

## 4. Conclusions

From the roots of *W. japonica*, a total 18 compounds (**1–18**), including a new phytochemical (**1**), were isolated by chromatographic methods and identified by spectroscopic and spectrometric data analysis, including 1D and 2D NMR and HRMS. The structure of the new compound **1** was assigned as a possibly dicarboxylic acid–derived 2-butenolide derivative and its biosynthetic pathway was also proposed based on the assigned structure and related literature search. Compound **1** showed strong anti-neuroinflammatory activity by inhibiting NO production in LPS-stimulated BV-2 cells and α-tocospiro A (**15**) exhibited a potent antiproliferative activity against A549 non-small cell lung adenocarcinoma cells.

## Figures and Tables

**Table 1:** Antiproliferative activities of hexanes-, CHCl_3_-, EtOAc-, and *n*-BuOH-soluble fractions of *W. japonica* MeOH extract against four cultured human cancer cell lines in the Sulforhodamine B (SRB) bioassay.

Fraction	GI_50_ (μg/mL) ^1^			
	A549	SK-OV-3	SK-MEL-2	HCT-15
hexanes	30.57	21.71	16.34	50.64
CHCl_3_	52.32	57.09	57.60	36.53
EtOAc	>100	>100	>100	>100
*n*-BuOH	>100	>100	40.01	>100

^1^ Half-maximal growth inhibitory concentration; the concentration of fraction that caused a 50% inhibition in cell growth.

**Table 2:** NMR spectroscopic data of compounds **1** and MEO measured in chloroform-*d*.

Pos.	1		MEO ^1^	
	δ_H_, Multi. (*J* in Hz)	δ_C_	δ_H_, Multi. (*J* in Hz)	δ_C_
1	-	170.1	-	169.98
2	6.22, d (5.7)	125.0	6.19, d (5.7)	124.75
3	7.12, d (5.7)	153.7	7.10, d (5.7)	153.53
4	-	111.4	-	111.28
5	1.89, m	37.1	1.90, m	36.98
6	1.37, m	23.3	1.23, brs; H-6–H-19	22.67–31.91; C-6–C-19
7	1.30, overlap	29.4 ^2^	0.86, t; H-20	14.10; C-20
8	1.30, overlap	29.07 ^2^	-	-
9	1.30, overlap	29.13 ^2^	-	-
10	1.61, m	25.0	-	-
11	2.29, t (7.5)	34.2	-	-
12	-	174.4	-	-
OCH_3_-4	3.22, s	51.3	3.20, s	51.11
OCH_3_-12	3.66, s	51.6	-	-

^1^ Adapted from the previous research [16]. ^2^ Exchangeable peaks.

**Table 3:** Antiproliferative activities of selected compounds against four cultured human cell lines.

Compound	IC_50_ (μM) ^1^			
	A549	SK-OV-3	SK-MEL-2	MKN-1
10	26.03	>30.0	>30.0	>30.0
11	17.95	>30.0	17.43	>30.0
14	13.28	12.86	13.04	14.17
15	2.10	13.23	9.08	10.04
16	19.21	>30.0	>30.0	>30.0
18	24.64	20.16	26.72	31.49
Etoposide ^2^	1.51	1.94	1.13	3.37

^1^ 50% inhibitory concentration; the concentration of compound that caused a 50% inhibition in cell growth. ^2^ Etoposide was used as a positive control.

**Table 4:** Effects of select compounds on NO generation in LPS-stimulated BV-2 cells.

Compound	IC_50_ (μM) ^1^	Cell Viability (%) ^2^
1	45.3	112.07 ± 4.78
5	90.0	98.31 ± 11.14
11	59.6	67.91 ± 4.06
15	92.4	120.77 ± 8.20
L-NMMA ^3^	21.4	104.56 ± 4.20

^1^ IC_50_ value of each compound was defined as the concentration (μM) that caused 50% inhibition of NO production in LPS-activated BV-2 cells. ^2^ Cell viability after treatment with 20 μM of each compound was determined by 3-[4,5-dimethylthiazol-2-yl]-2,5-diphenyltetrazolium bromide (MTT) assay and is expressed in percentage (%). The results are averages of three independent experiments, and the data are expressed as mean ± SD. ^3^ *N*^G^-monomethyl-l-arginine (l-NMMA) was used as a positive control.

## Data Availability

Data is contained within the article or Supplementary Materials.

## Supplementary Materials

The following are available online at https://www.mdpi.com/article/10.3390/antiox11030482/s1. Figure S1: HRESIMS spectrum of **1**. Figures S2–S6: 1D and 2D NMR spectra of **1**. Figures S7–S34: ^1^H and/or ^13^C NMR spectra of **2**–**18**.
