# Supplementary material for: Anticancer and Anti-Neuroinflammatory Constituents Isolated from the Roots of Wasabia japonica
Source: Antioxidants (Basel). 2022 Feb 28;11(3):482. doi: 10.3390/antiox11030482 (PMC8944812; doi:10.3390/antiox11030482)

# Anticancer and Anti-neuroinflammatory Constituents Isolated from the Roots of *Wasabia japonica*

Jong Eel Park [1,2], Tae Hyun Lee [1,3], Song Lim Ham [3], Lalita Subedi [4], Seong Min Hong [4], Sun Yeou Kim [4,5], Sang Un Choi [6], Chung Sub Kim [1,3,*] and Kang Ro Lee [1,*]

[1]School of Pharmacy, Sungkyunkwan University, Suwon 16419, Republic of Korea

[2]Korea Environment Corporation, 42 Hwangyeong-ro, Seo-gu, Incheon 22689, Republic of Korea

[3]Department of Biopharmaceutical Convergence, Sungkyunkwan University, Suwon, 16419, Republic of Korea

[4]Gachon Institute of Pharmaceutical Science, Gachon University, Incheon 21936, Republic of Korea

[5]College of Pharmacy, Gachon University, #191, Hambakmoero, Yeonsu-gu, Incheon 21936, Republic of Korea

[6]Korea Research Institute of Chemical Technology, Daejeon 34114, Republic of Korea

# Contents


**Figure S1.** HRESIMS spectrum of **1** ........ 1
**Figure S2.** $^1$H NMR spectrum of **1** in chloroform-*d* (700 MHz) ........ 2
**Figure S3.** $^{13}$C NMR spectrum of **1** in chloroform-*d* (175 MHz) ........ 3
**Figure S4.** COSY spectrum of **1** in chloroform-*d* ........ 4
**Figure S5.** HSQC spectrum of **1** in chloroform-*d* ........ 5
**Figure S6.** HMBC spectrum of **1** in chloroform-*d* ........ 6
**Figure S7.** $^1$H NMR spectrum of **2** in chloroform-*d* (700 MHz) ........ 7
**Figure S8.** $^{13}$C NMR spectrum of **2** in chloroform-*d* (175 MHz) ........ 8
**Figure S9.** $^1$H NMR spectrum of **3** in chloroform-*d* (700 MHz) ........ 9
**Figure S10.** $^1$H NMR spectrum of **4** in methanol-$d_4$ (700 MHz) ........ 10
**Figure S11.** $^1$H NMR spectrum of **5** in chloroform-*d* (700 MHz) ........ 11
**Figure S12.** $^{13}$C NMR spectrum of **5** in chloroform-*d* (175 MHz) ........ 12
**Figure S13.** $^1$H NMR spectrum of **6** in methanol-$d_4$ (700 MHz) ........ 13
**Figure S14.** $^{13}$C NMR spectrum of **6** in methanol-$d_4$ (175 MHz) ........ 14
**Figure S14.** $^1$H NMR spectrum of **7** in methanol-$d_4$ (700 MHz) ........ 15
**Figure S15.** $^{13}$C NMR spectrum of **7** in methanol-$d_4$ (175 MHz) ........ 16
**Figure S16.** $^1$H NMR spectrum of **8** in chloroform-*d* (700 MHz) ........ 17
**Figure S17.** $^1$H NMR spectrum of **9** in chloroform-*d* (700 MHz) ........ 18
**Figure S18.** $^1$H NMR spectrum of **10** in chloroform-*d* (700 MHz) ........ 19
**Figure S19.** $^{13}$C NMR spectrum of **10** in chloroform-*d* (175 MHz) ........ 20
**Figure S20.** $^1$H NMR spectrum of **11** in chloroform-*d* (700 MHz) ........ 21
**Figure S21.** $^{13}$C NMR spectrum of **11** in chloroform-*d* (175 MHz) ........ 22
**Figure S22.** $^1$H NMR spectrum of **12** in chloroform-*d* (700 MHz) ........ 23
**Figure S23.** $^{13}$C NMR spectrum of **12** in chloroform-*d* (175 MHz) ........ 24
**Figure S24.** $^1$H NMR spectrum of **13** in chloroform-*d* (700 MHz) ........ 25
**Figure S25.** $^1$H NMR spectrum of **14** in methanol-$d_4$ (700 MHz) ........ 26
**Figure S26.** $^{13}$C NMR spectrum of **14** in methanol-$d_4$ (175 MHz) ........ 27
**Figure S27.** $^1$H NMR spectrum of **15** in chloroform-*d* (700 MHz) ........ 28
**Figure S28.** $^{13}$C NMR spectrum of **15** in chloroform-*d* (175 MHz) ........ 29
**Figure S29.** $^1$H NMR spectrum of **16** in chloroform-*d* (700 MHz) ........ 30
**Figure S30.** $^{13}$C NMR spectrum of **16** in chloroform-*d* (175 MHz) ........ 31
**Figure S31.** $^1$H NMR spectrum of **17** in chloroform-*d* (700 MHz) ........ 32
**Figure S32.** $^{13}$C NMR spectrum of **17** in chloroform-*d* (175 MHz) ........ 33
**Figure S33.** $^1$H NMR spectrum of **18** in chloroform-*d* (700 MHz) ........ 34
**Figure S34.** $^{13}$C NMR spectrum of **18** in chloroform-*d* (175 MHz) ........ 35

**Figure S1.** HRESIMS spectrum of **1**

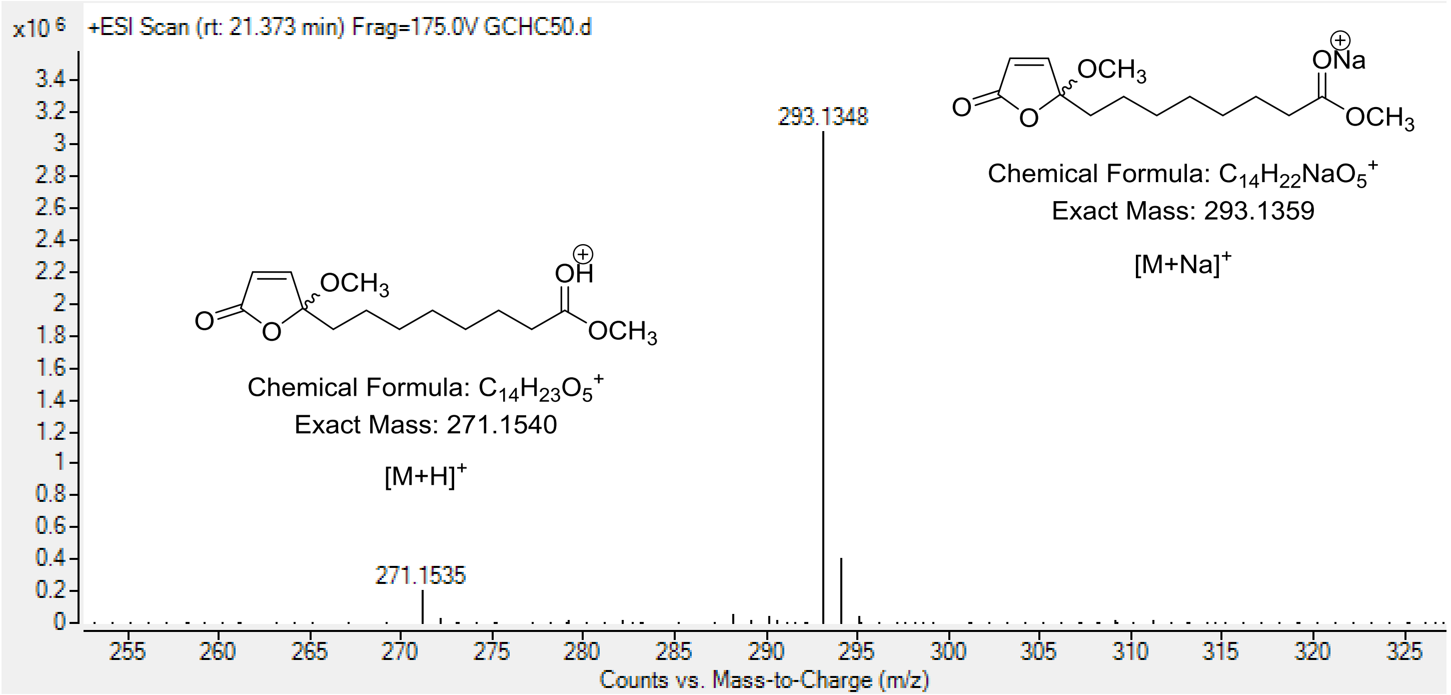

**Figure S2.** $^{1}$H NMR spectrum of **1** in chloroform-*d* (700 MHz)

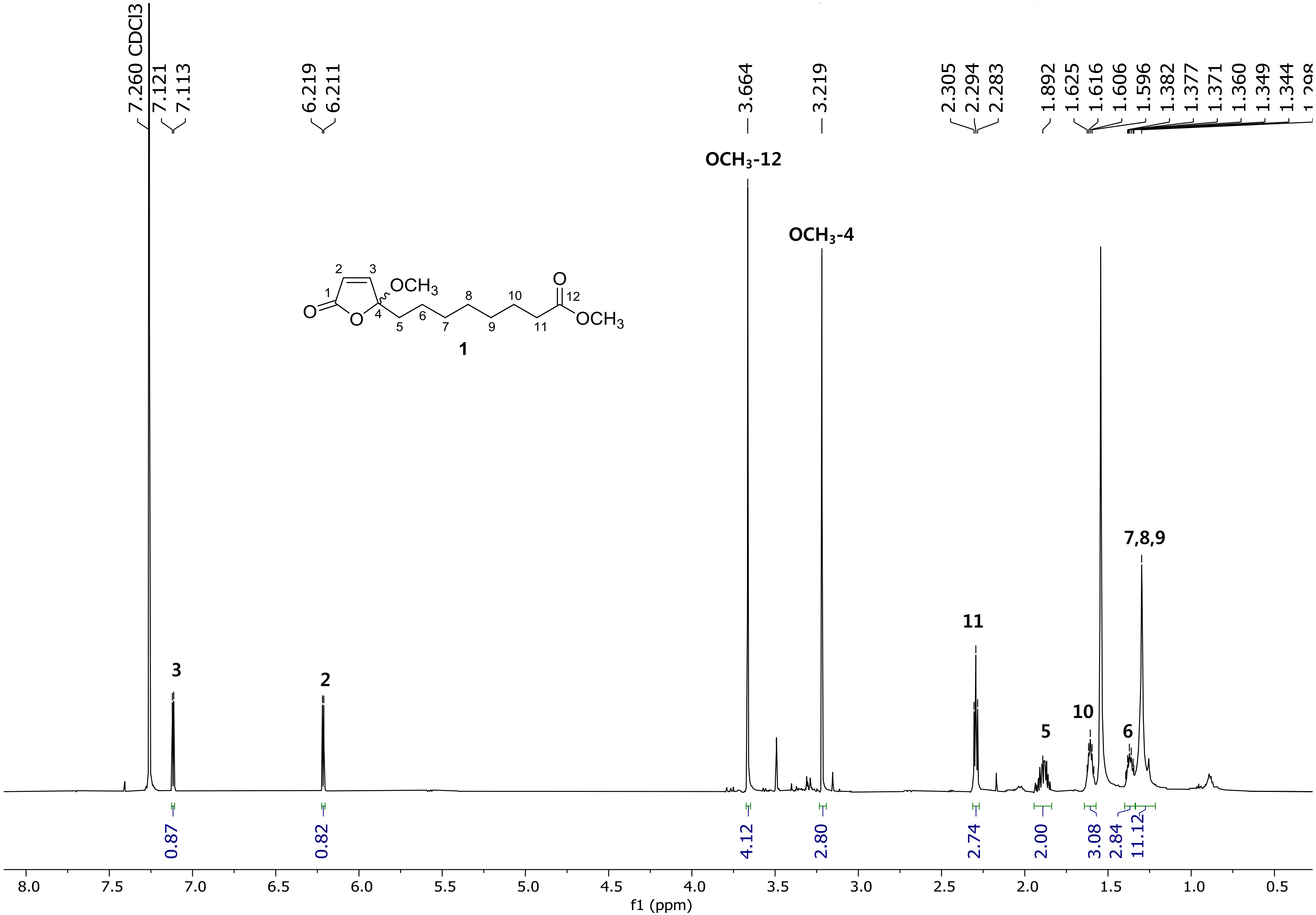

**Figure S3.** $^{13}$C NMR spectrum of **1** in chloroform-*d* (175 MHz)

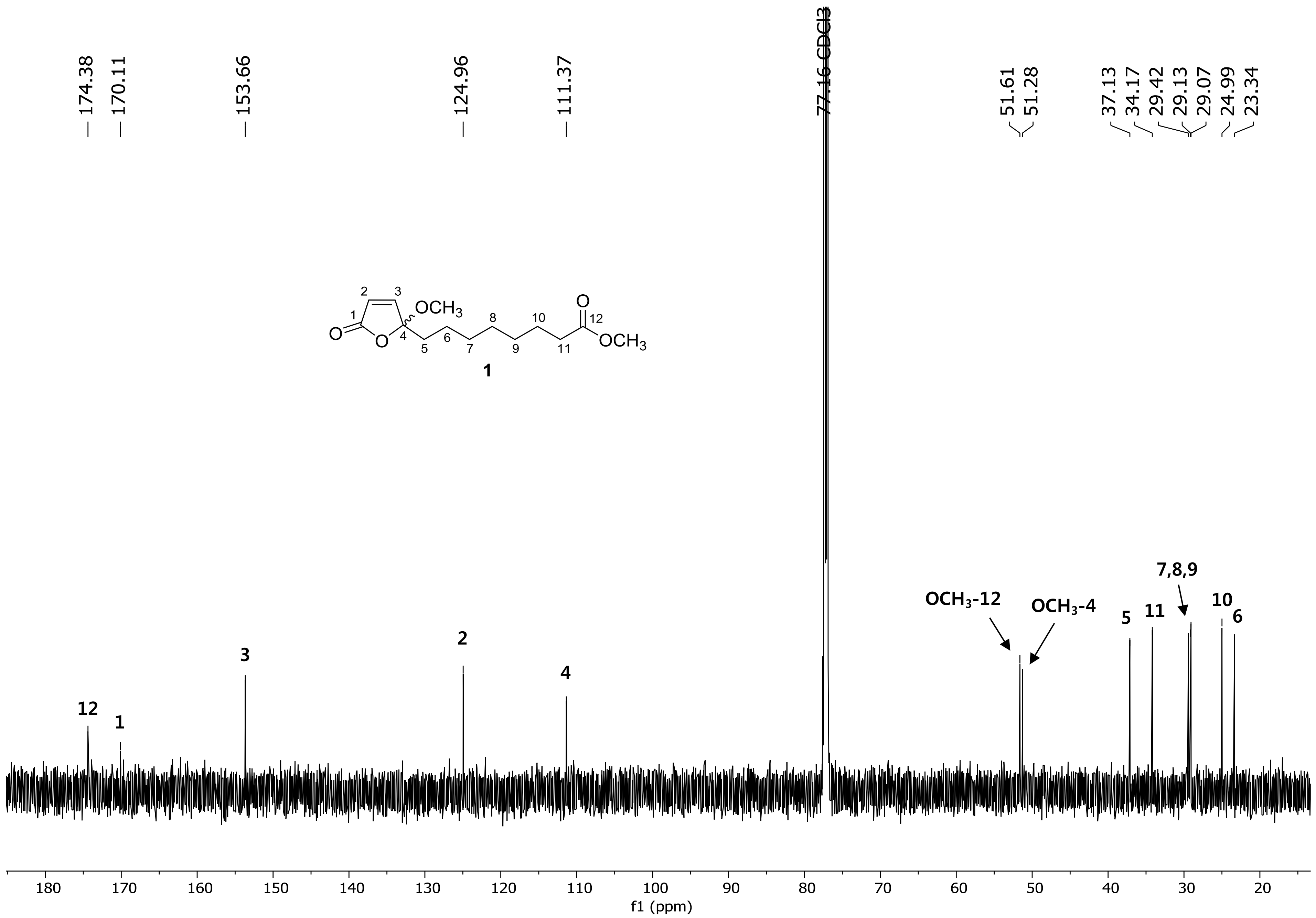

**Figure S4.** COSY spectrum of **1** in chloroform-*d*

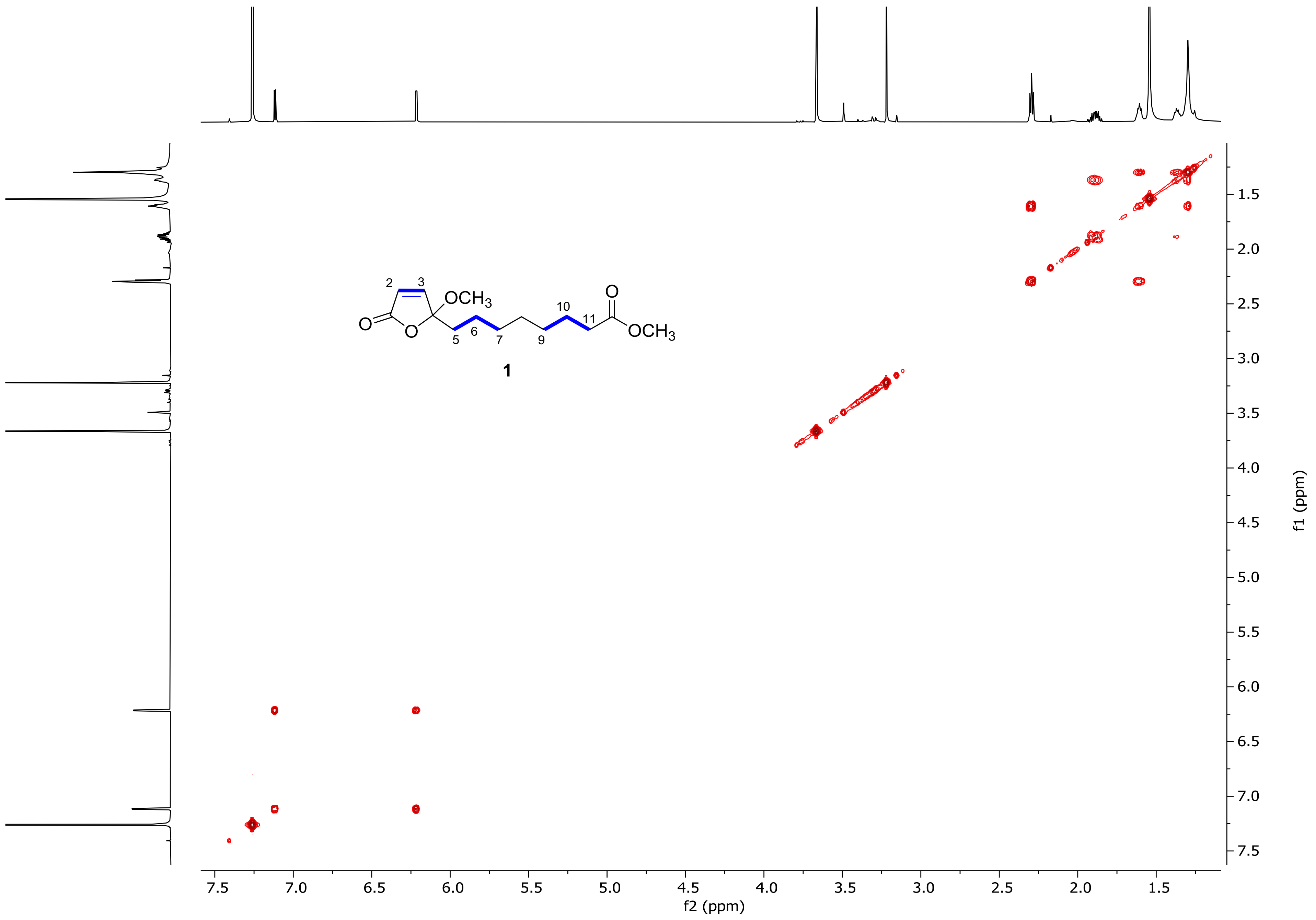

**Figure S5.** HSQC spectrum of **1** in chloroform-*d*

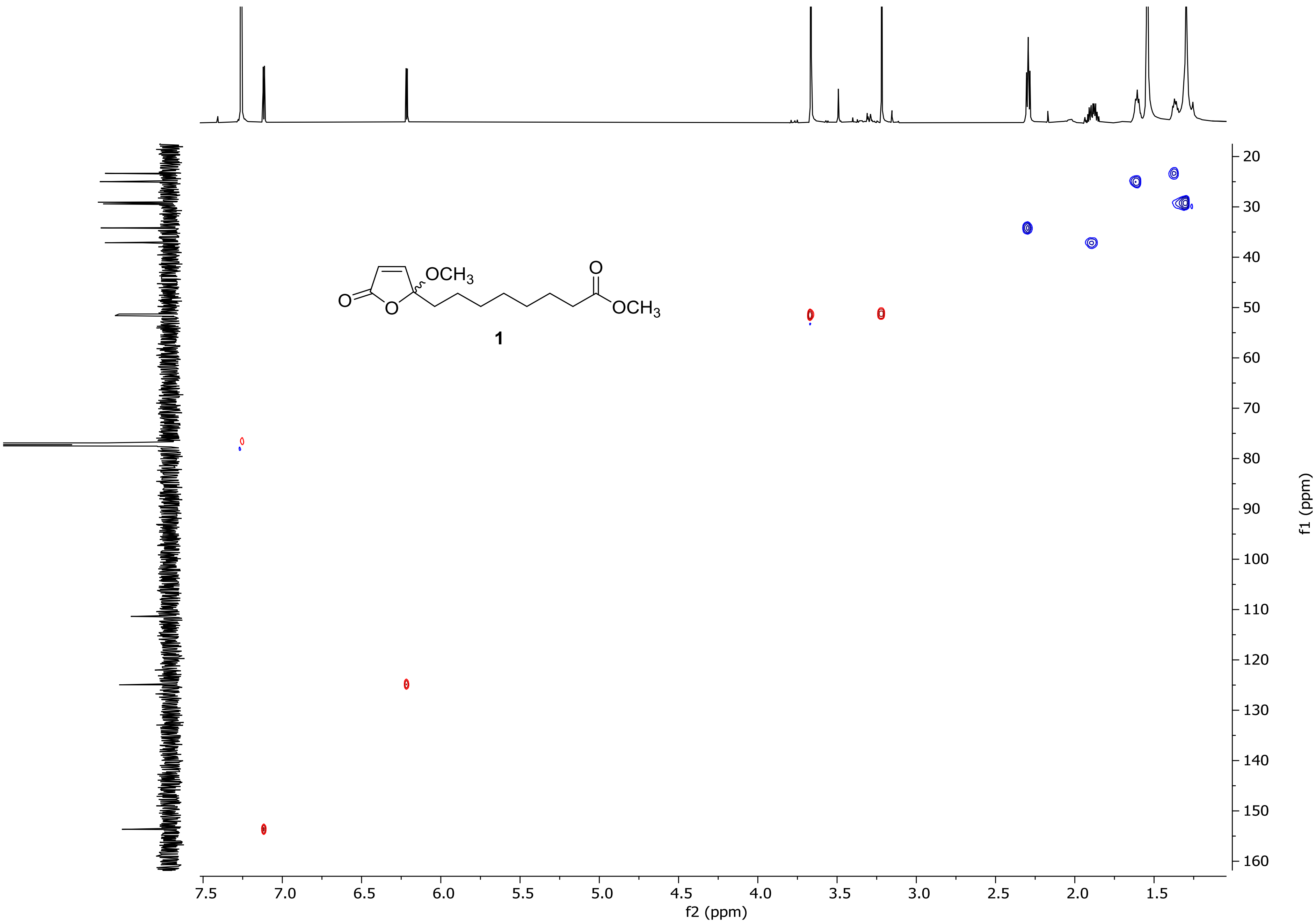

**Figure S6.** HMBC spectrum of **1** in chloroform-*d*

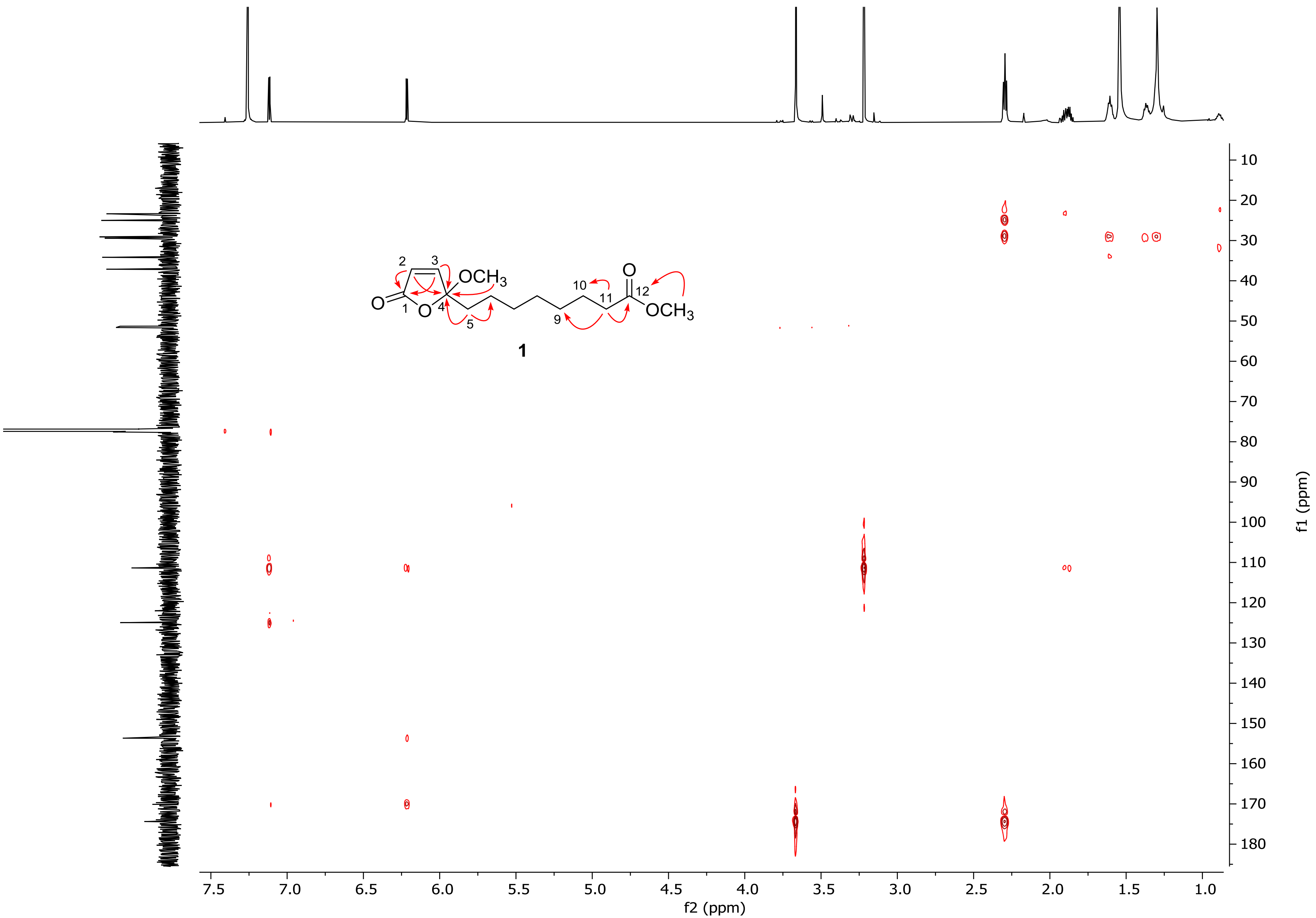

**Figure S7.** $^{1}$H NMR spectrum of **2** in chloroform-*d* (700 MHz)

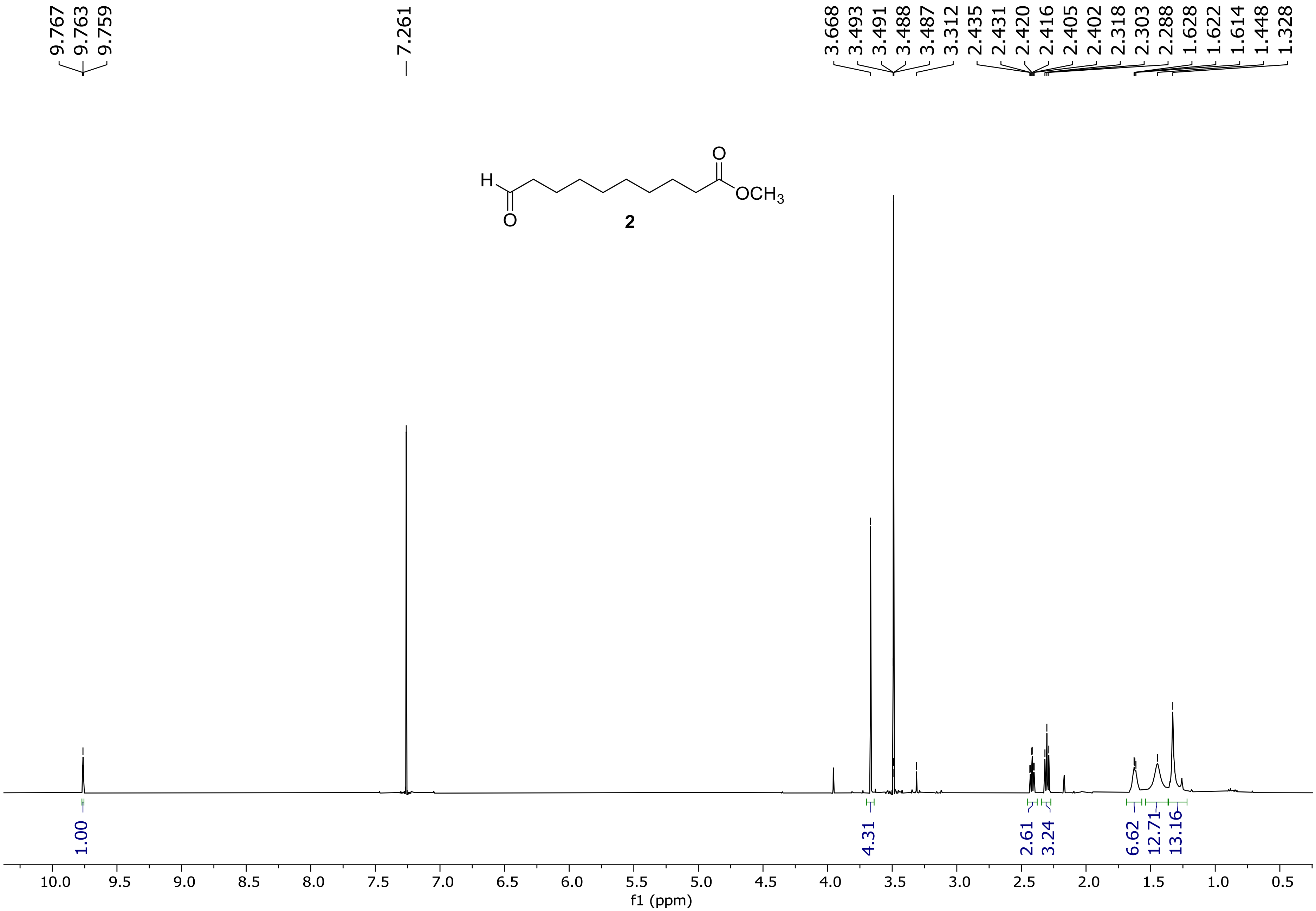

**Figure S8.** $^{13}C$ NMR spectrum of **2** in chloroform-*d* (175 MHz)

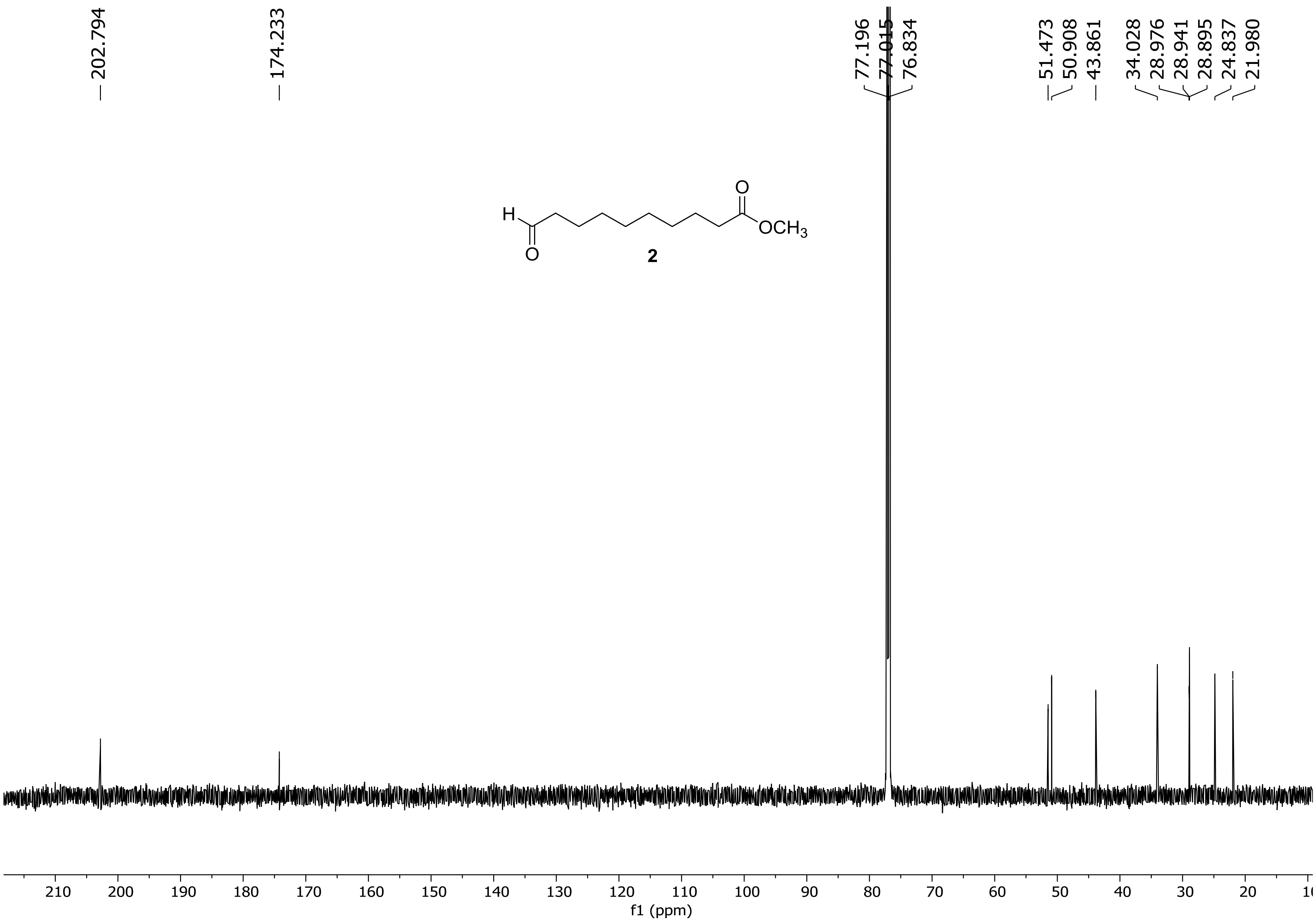

**Figure S9.** $^1$H NMR spectrum of **3** in chloroform-*d* (700 MHz)

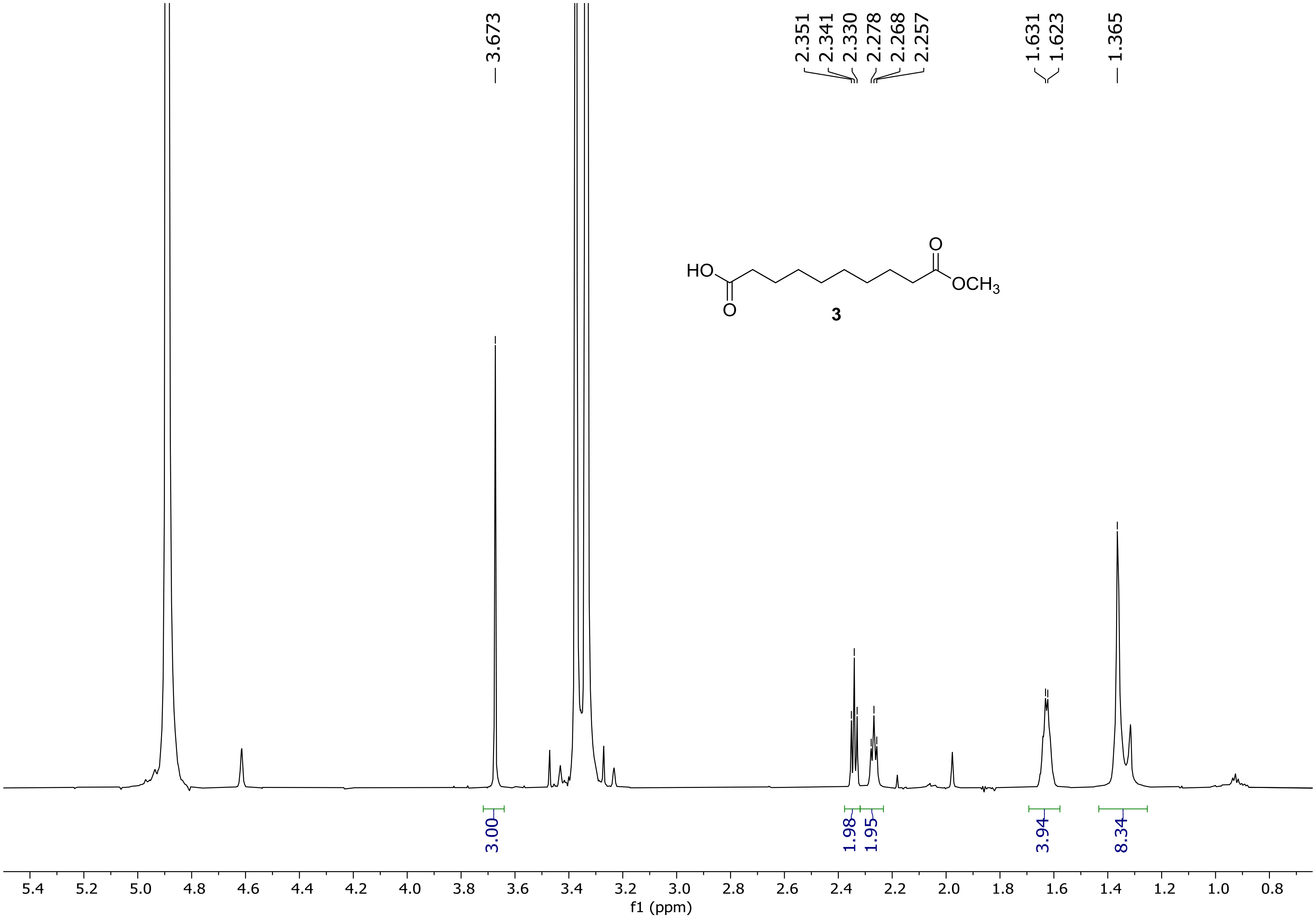

**Figure S10.** $^{1}$H NMR spectrum of **4** in methanol-$d_4$ (700 MHz)

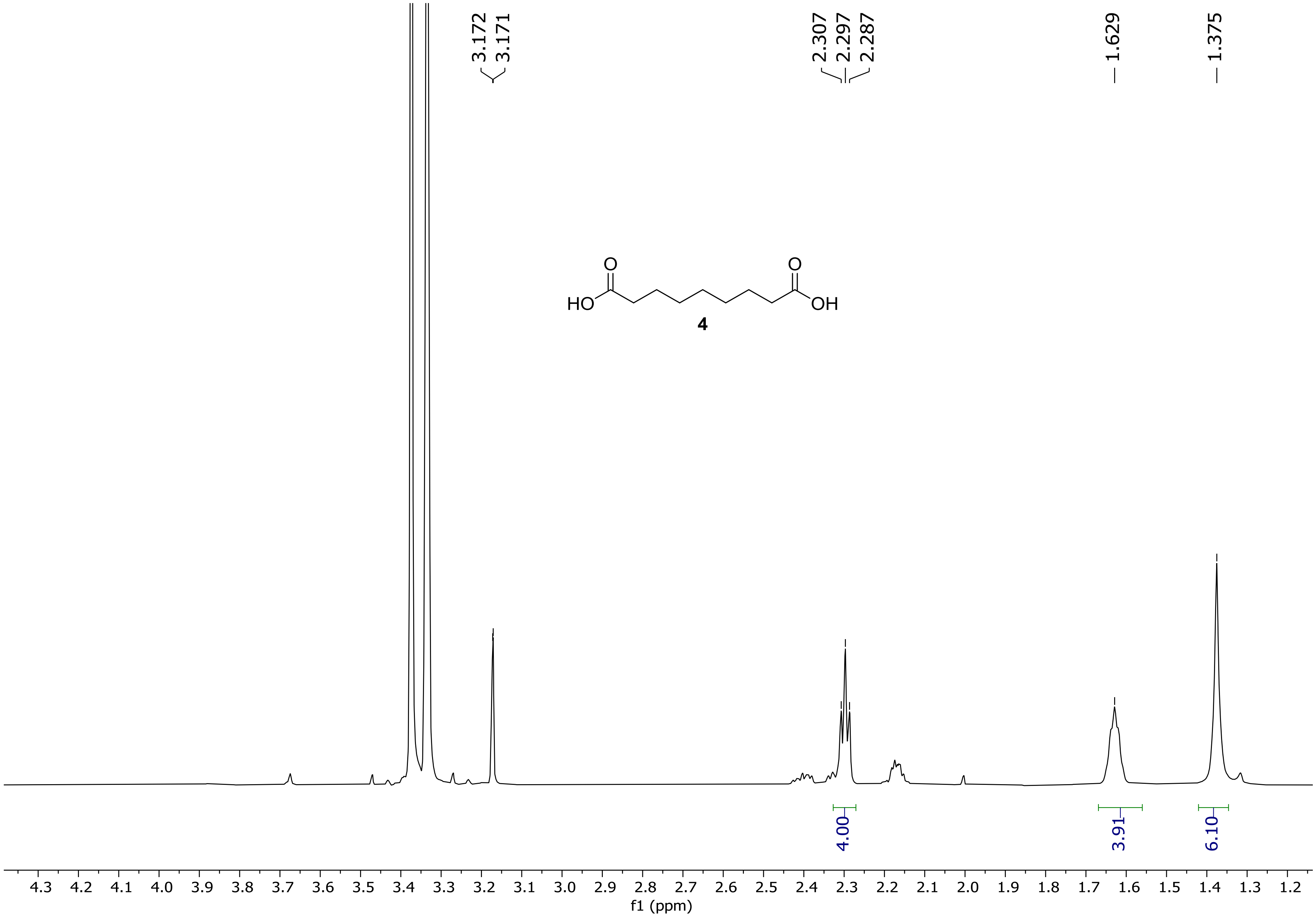

**Figure S11.** $^{1}$H NMR spectrum of **5** in chloroform-*d* (700 MHz)

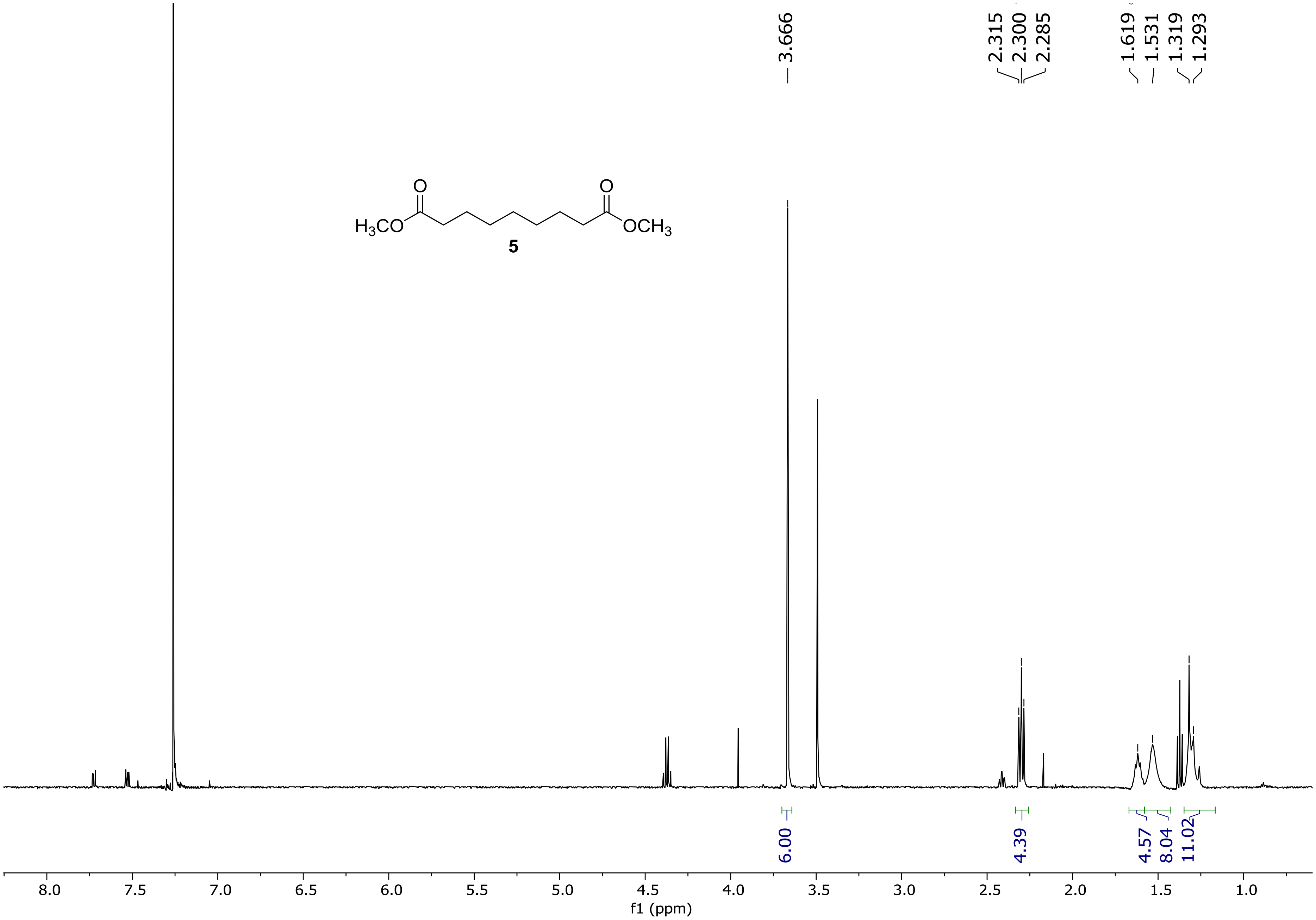

**Figure S12.** $^{13}C$ NMR spectrum of **5** in chloroform-*d* (175 MHz)

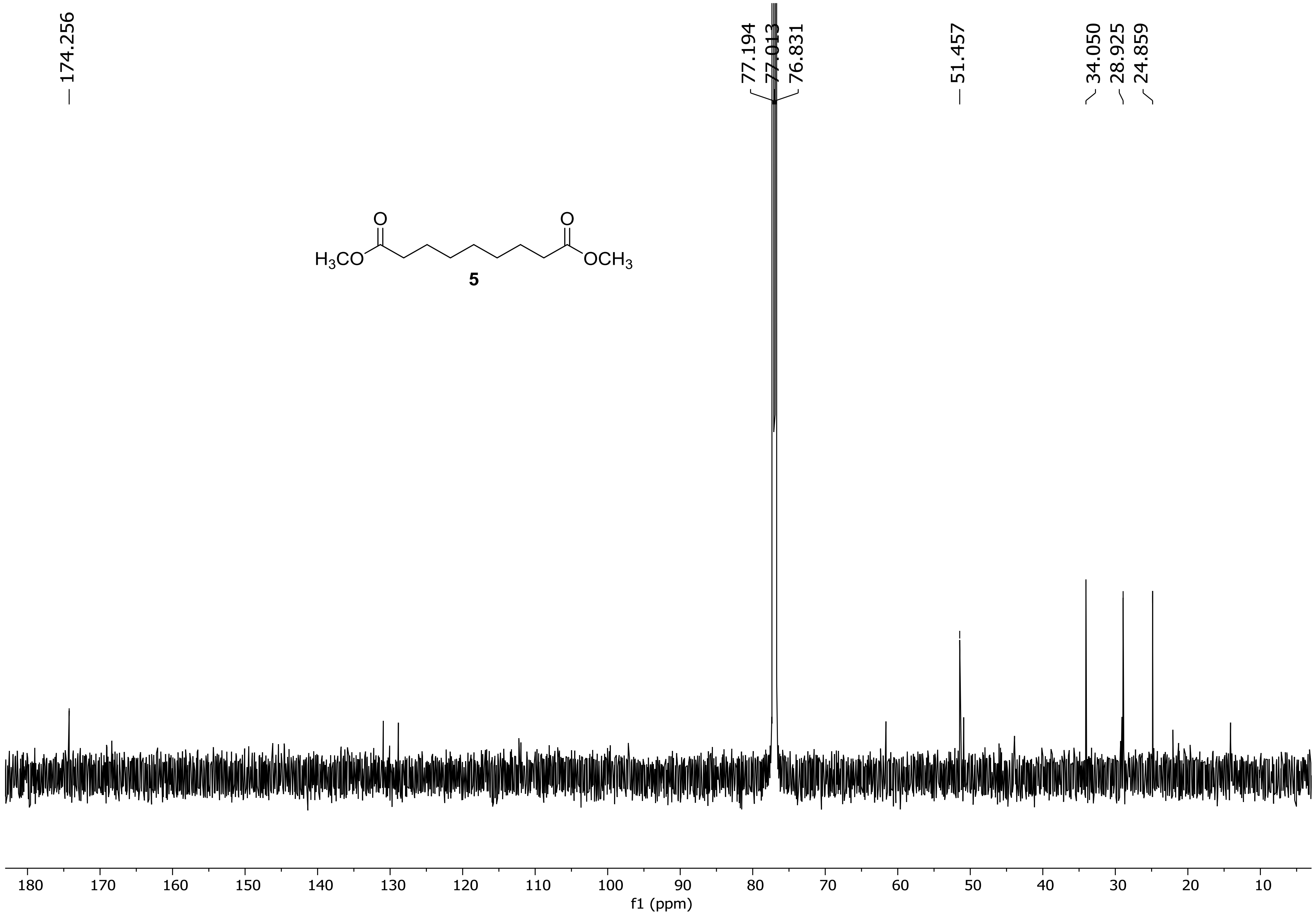

**Figure S13.** $^{1}$H NMR spectrum of **6** in methanol-$d_4$ (700 MHz)

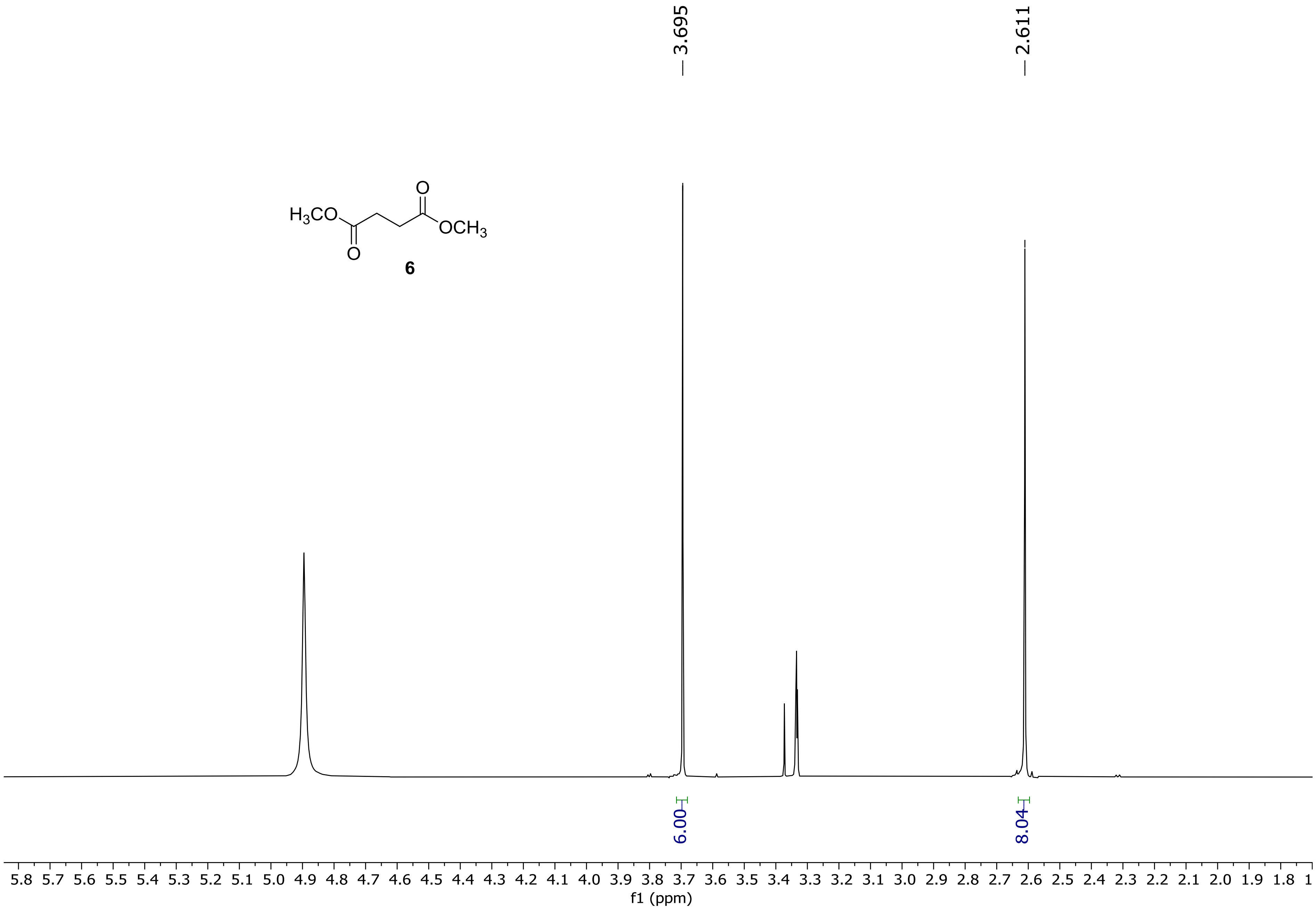

**Figure S14.** $^{13}C$ NMR spectrum of **6** in methanol-$d_4$ (175 MHz)

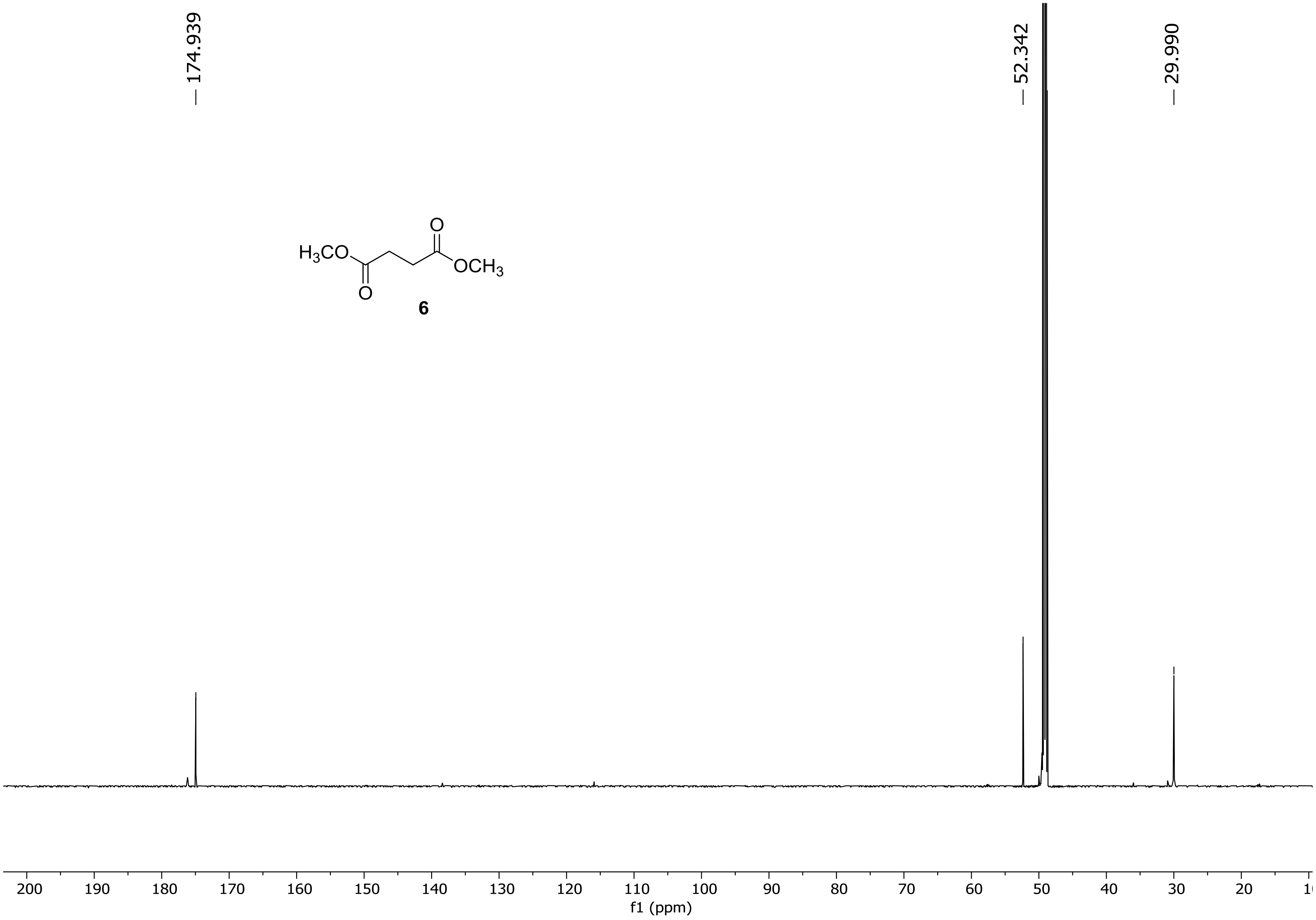

**Figure S15.** $^{1}$H NMR spectrum of **7** in methanol-$d_4$ (700 MHz)

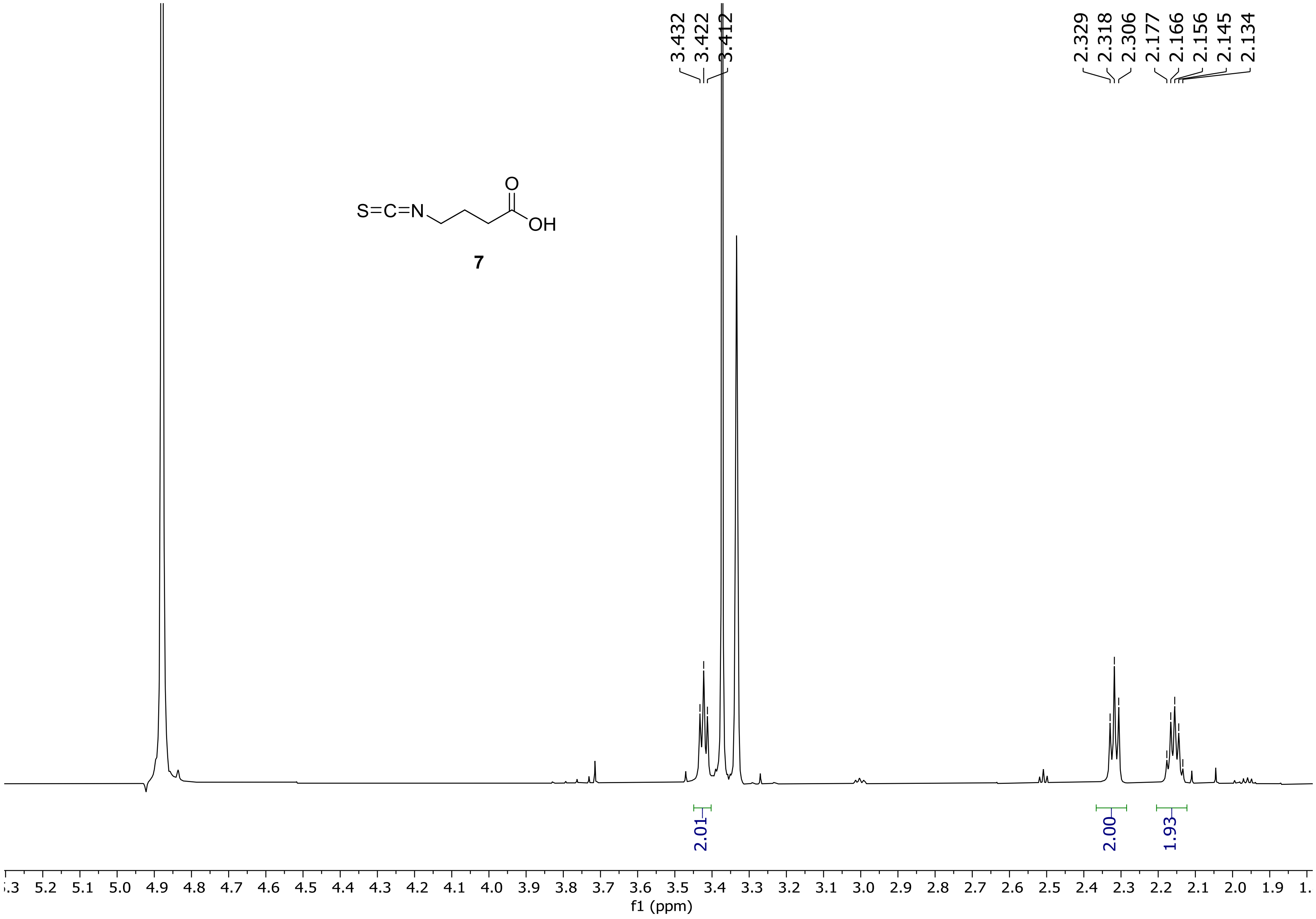

**Figure S16.** $^{13}$C NMR spectrum of **7** in methanol-$d_4$ (175 MHz)

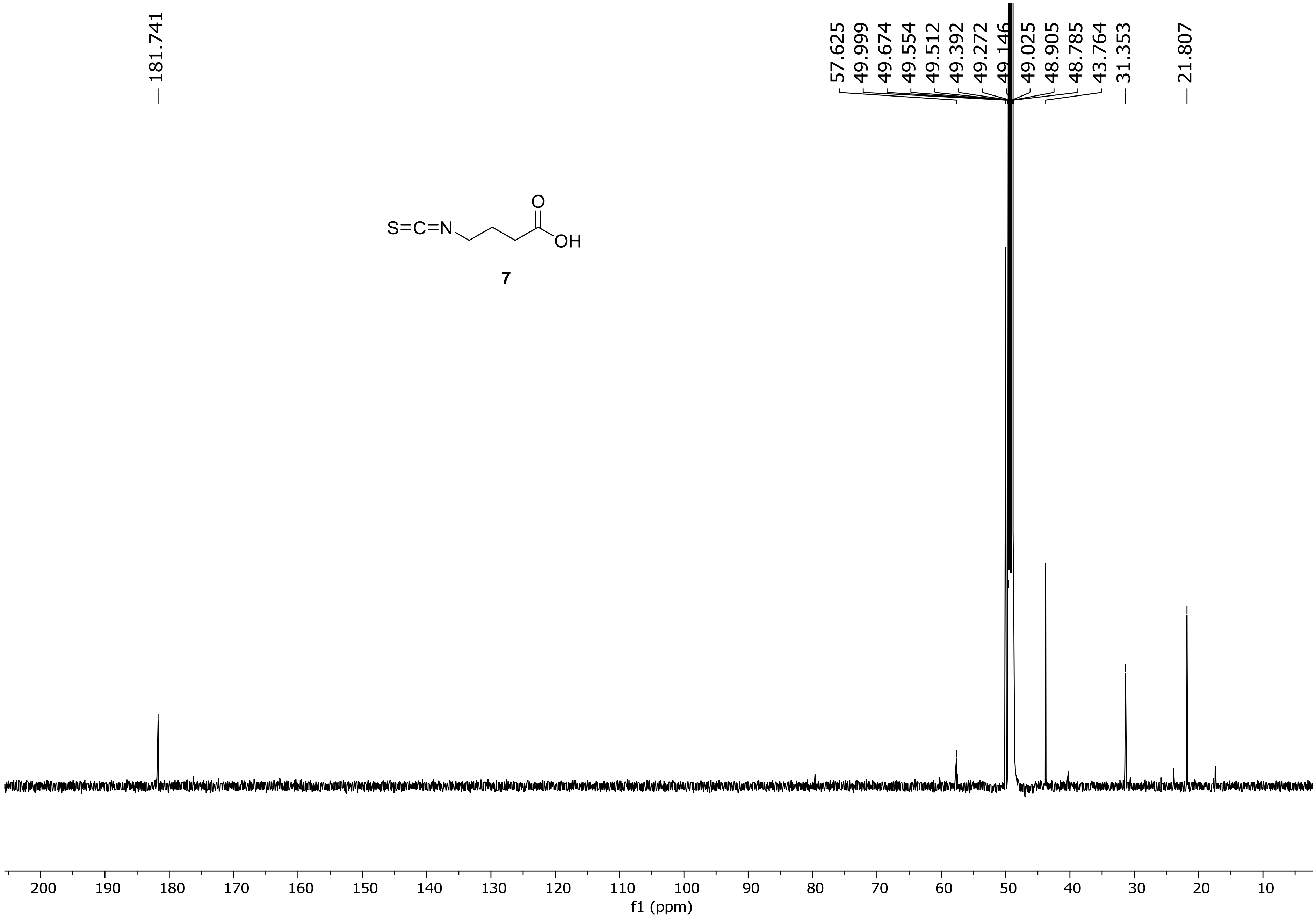

**Figure S17.** $^{1}$H NMR spectrum of **8** in chloroform-*d* (700 MHz)

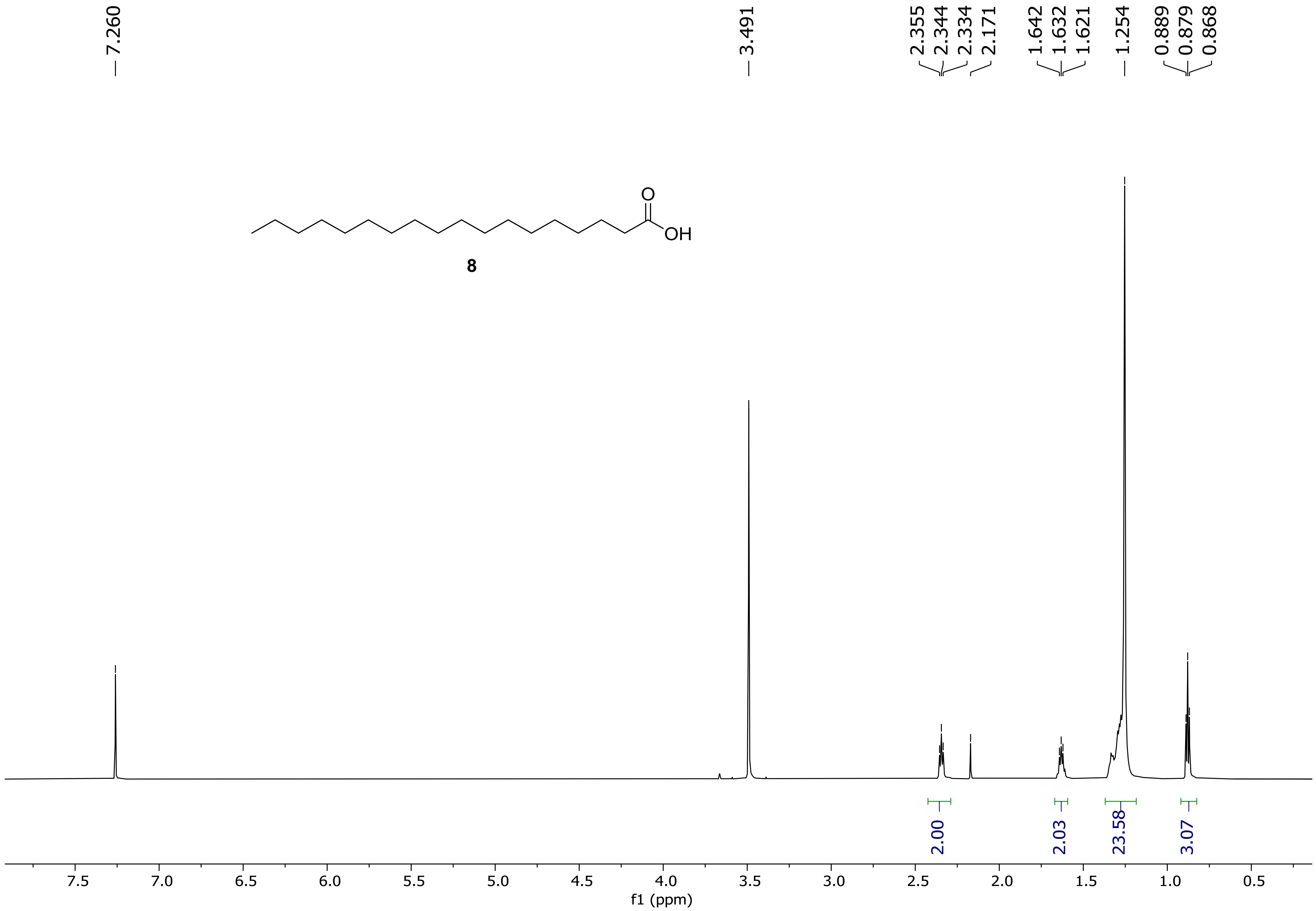

**Figure S18.** $^{1}$H NMR spectrum of **9** in chloroform-*d* (700 MHz)

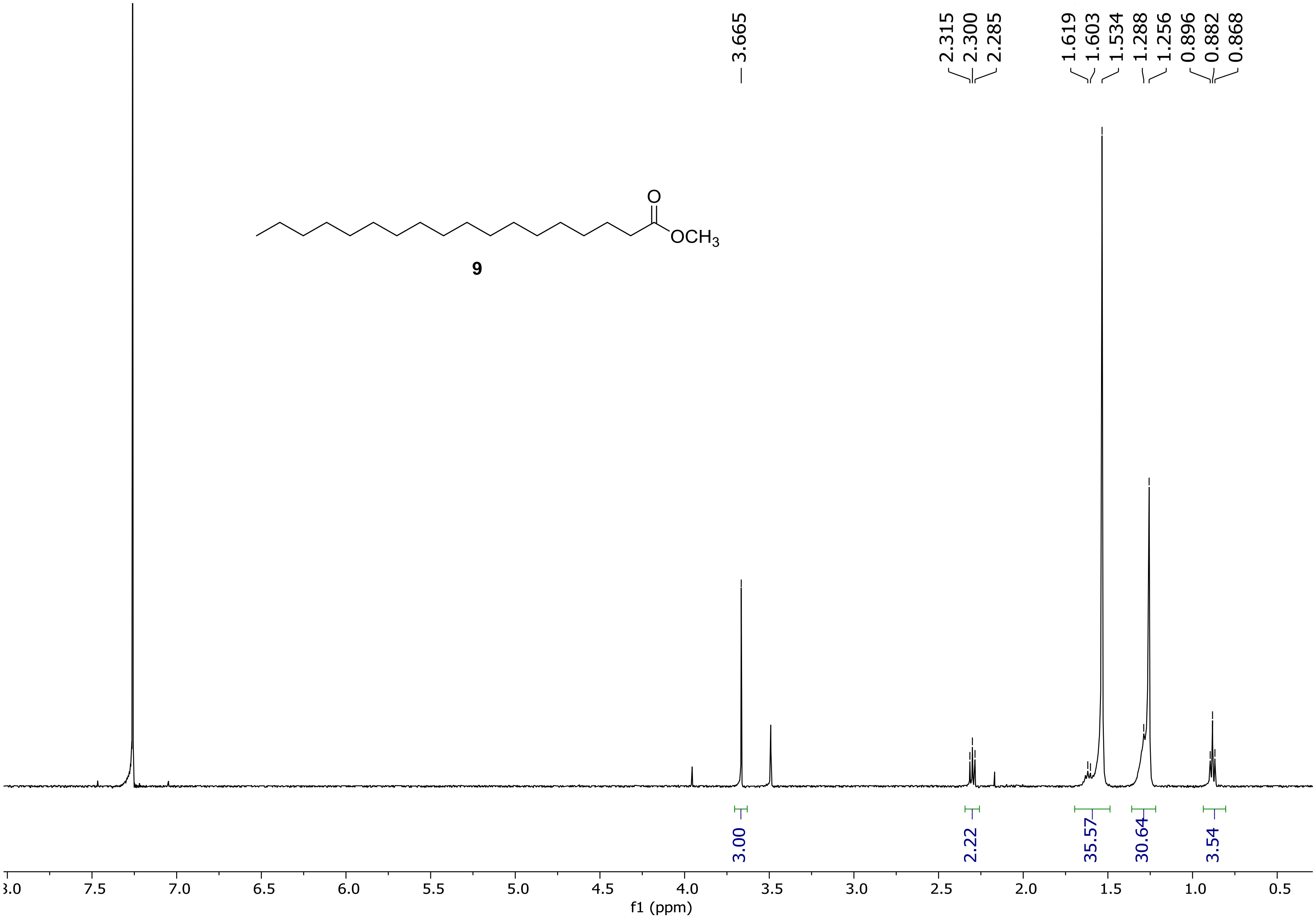

**Figure S19.** $^1$H NMR spectrum of **10** in chloroform-*d* (700 MHz)

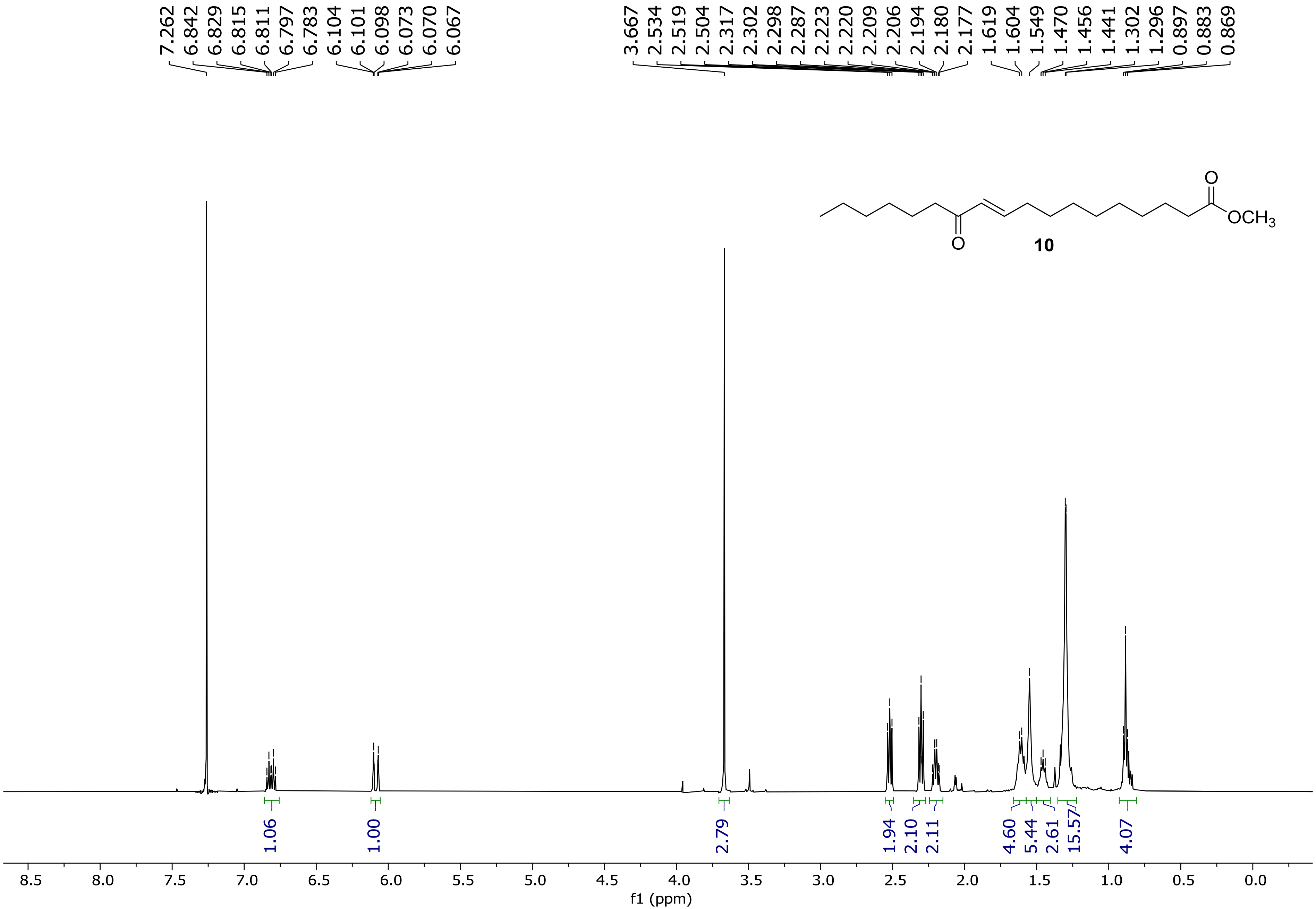

**Figure S20.** $^{13}C$ NMR spectrum of **10** in chloroform-*d* (175 MHz)

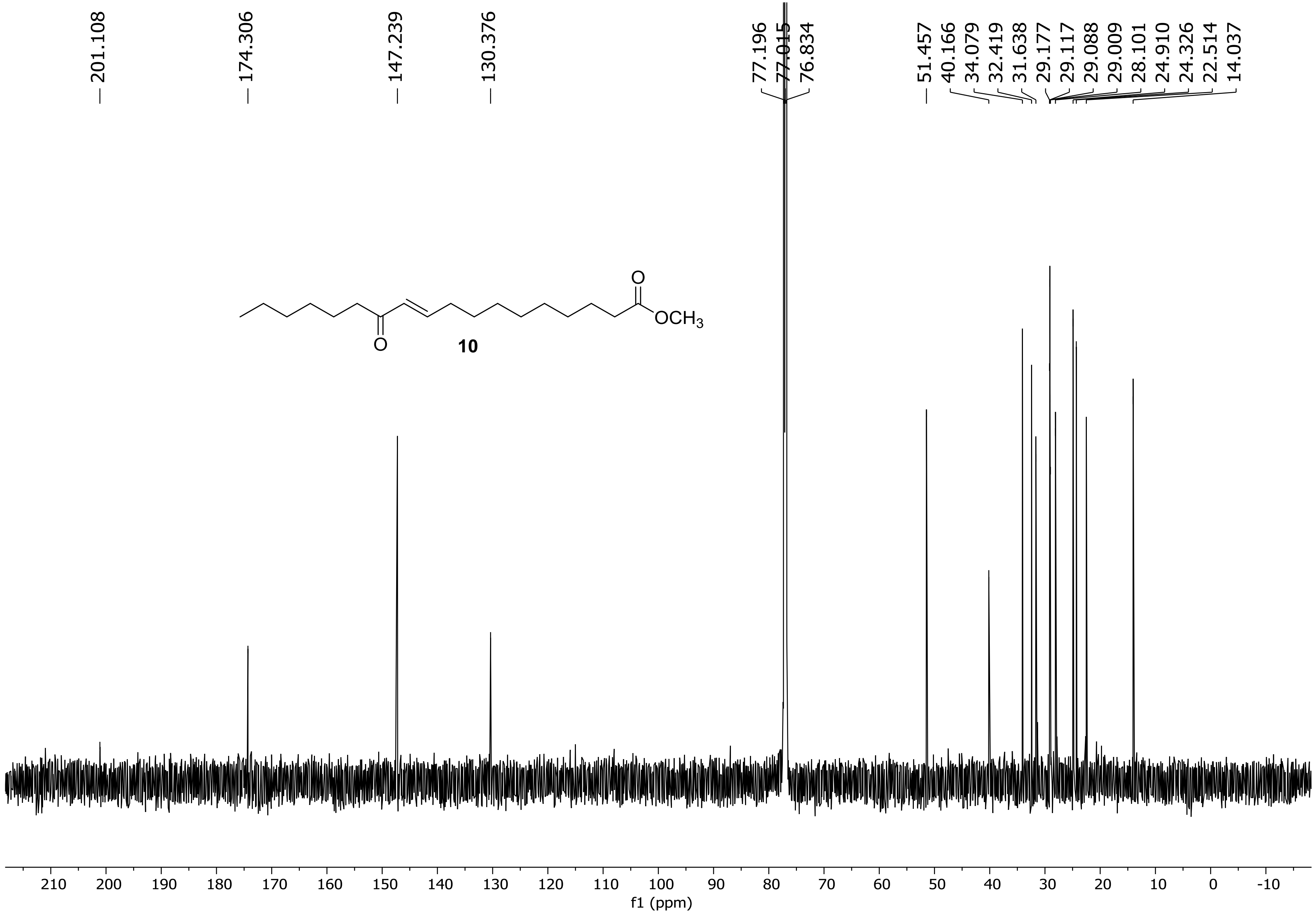

**Figure S21.** $^{1}$H NMR spectrum of **11** in chloroform-*d* (700 MHz)

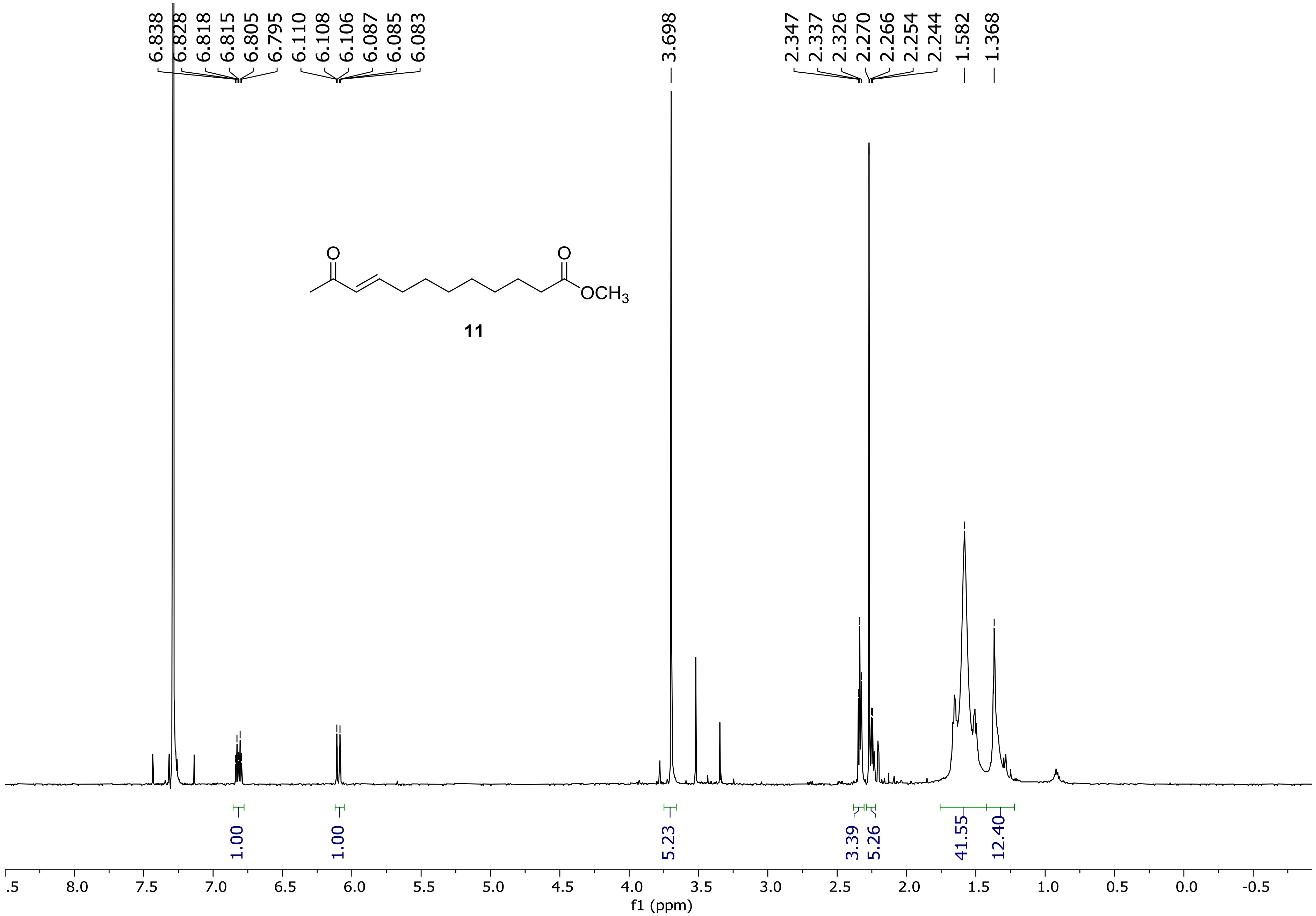

**Figure S22.** $^{13}$C NMR spectrum of **11** in chloroform-*d* (175 MHz)

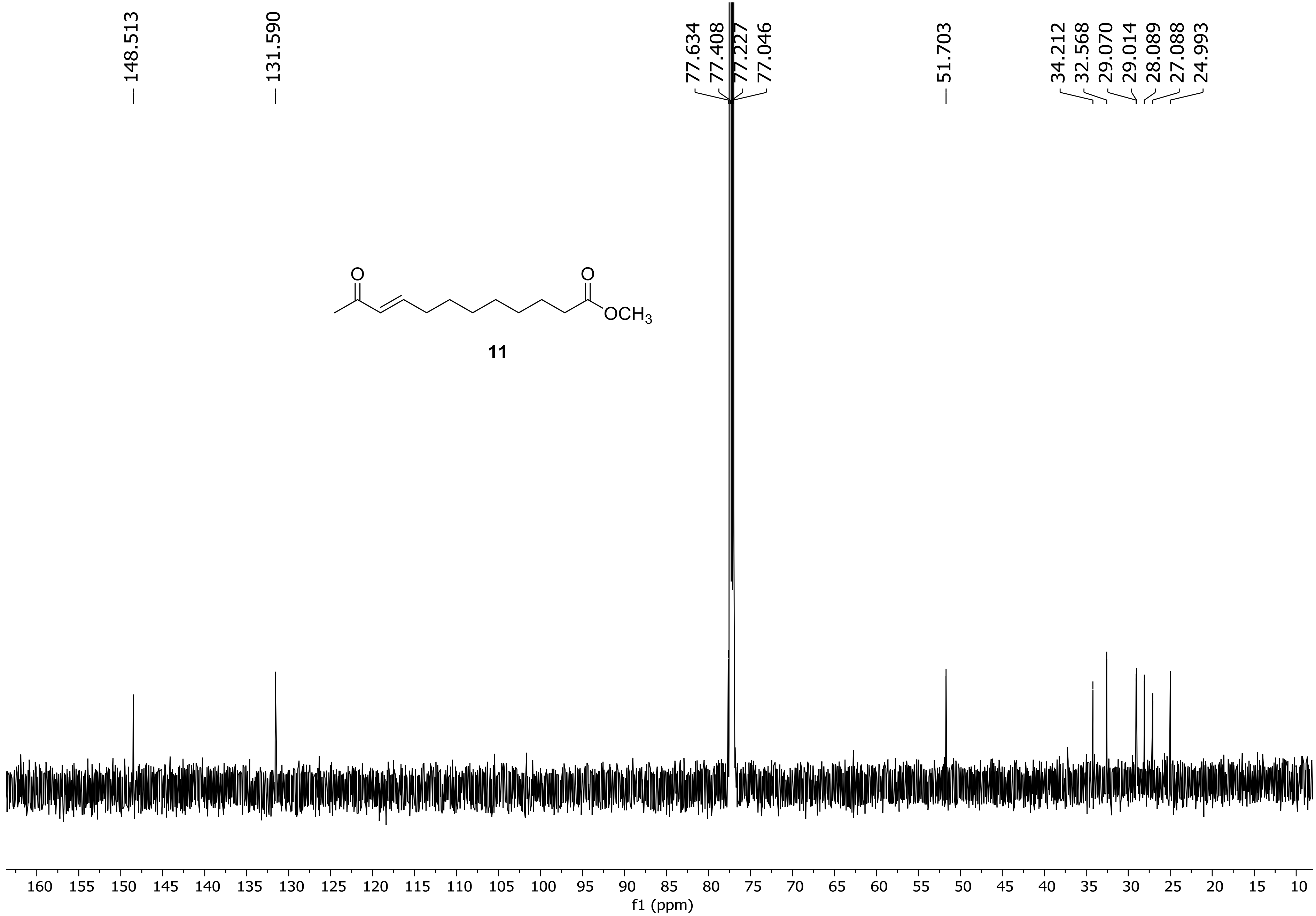

**Figure S23.** $^{1}$H NMR spectrum of **12** in chloroform-*d* (700 MHz)

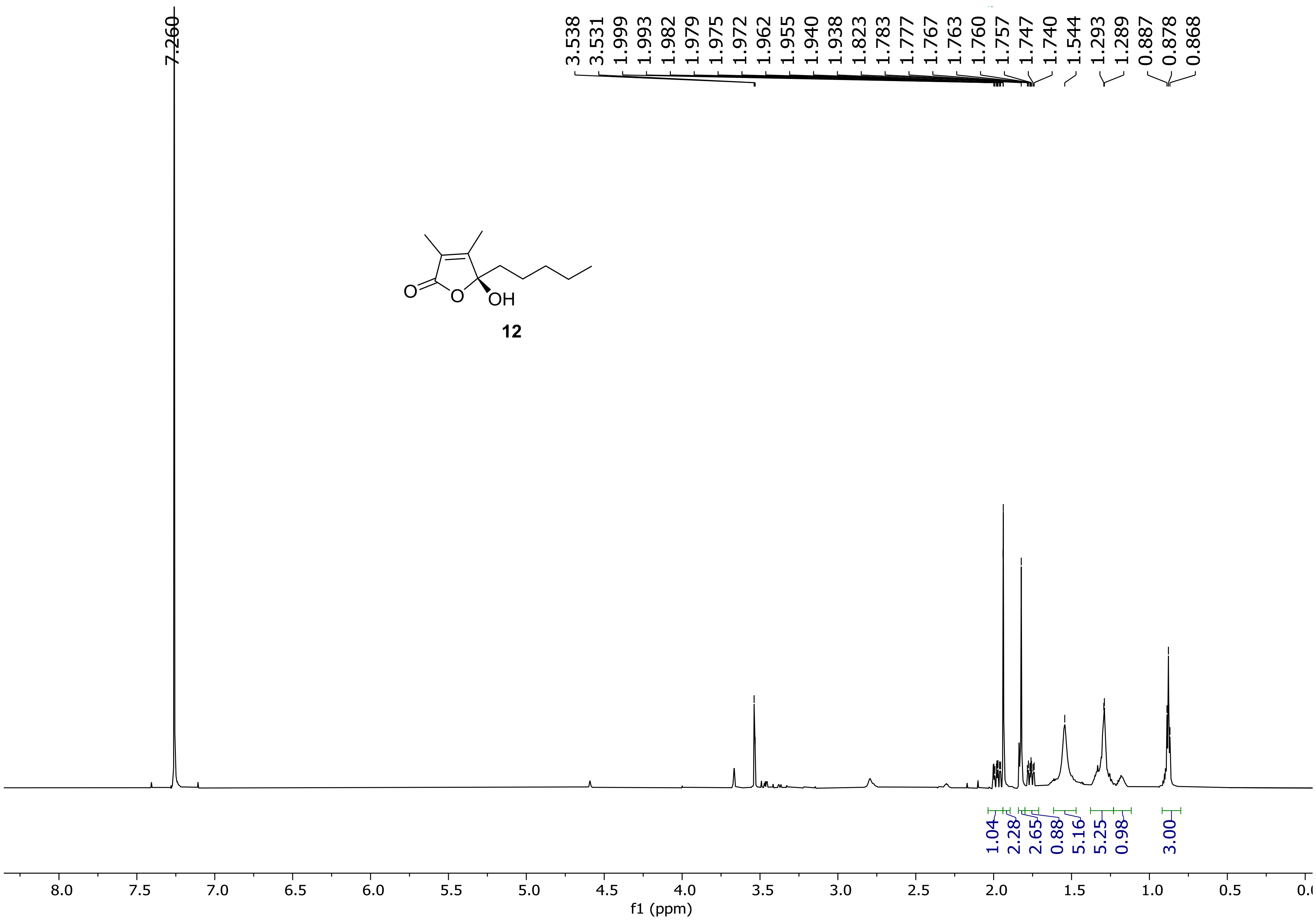

**Figure S24.** $^{13}$C NMR spectrum of **12** in chloroform-*d* (175 MHz)

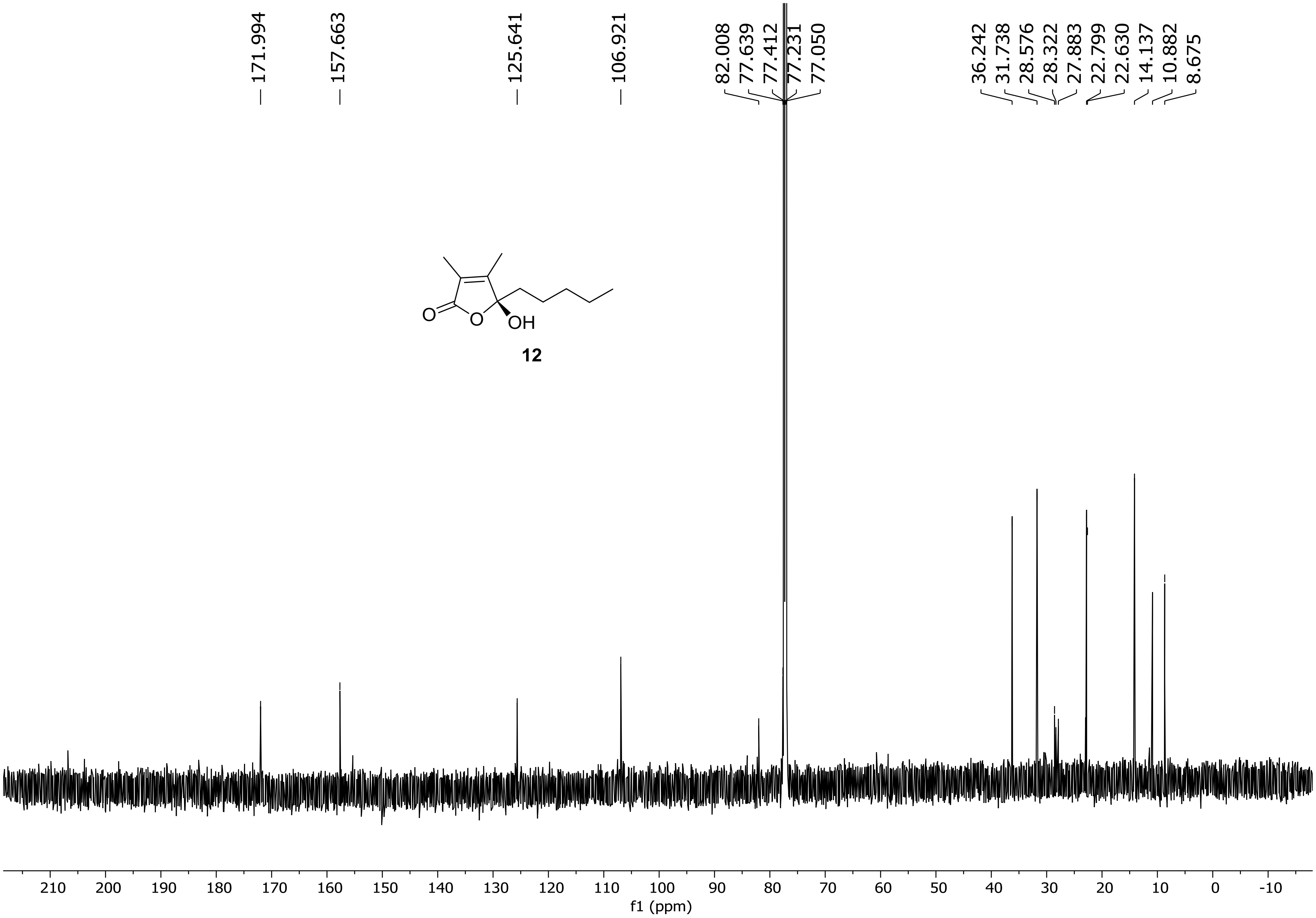

**Figure S25.** $^{1}$H NMR spectrum of **13** in chloroform-*d* (700 MHz)

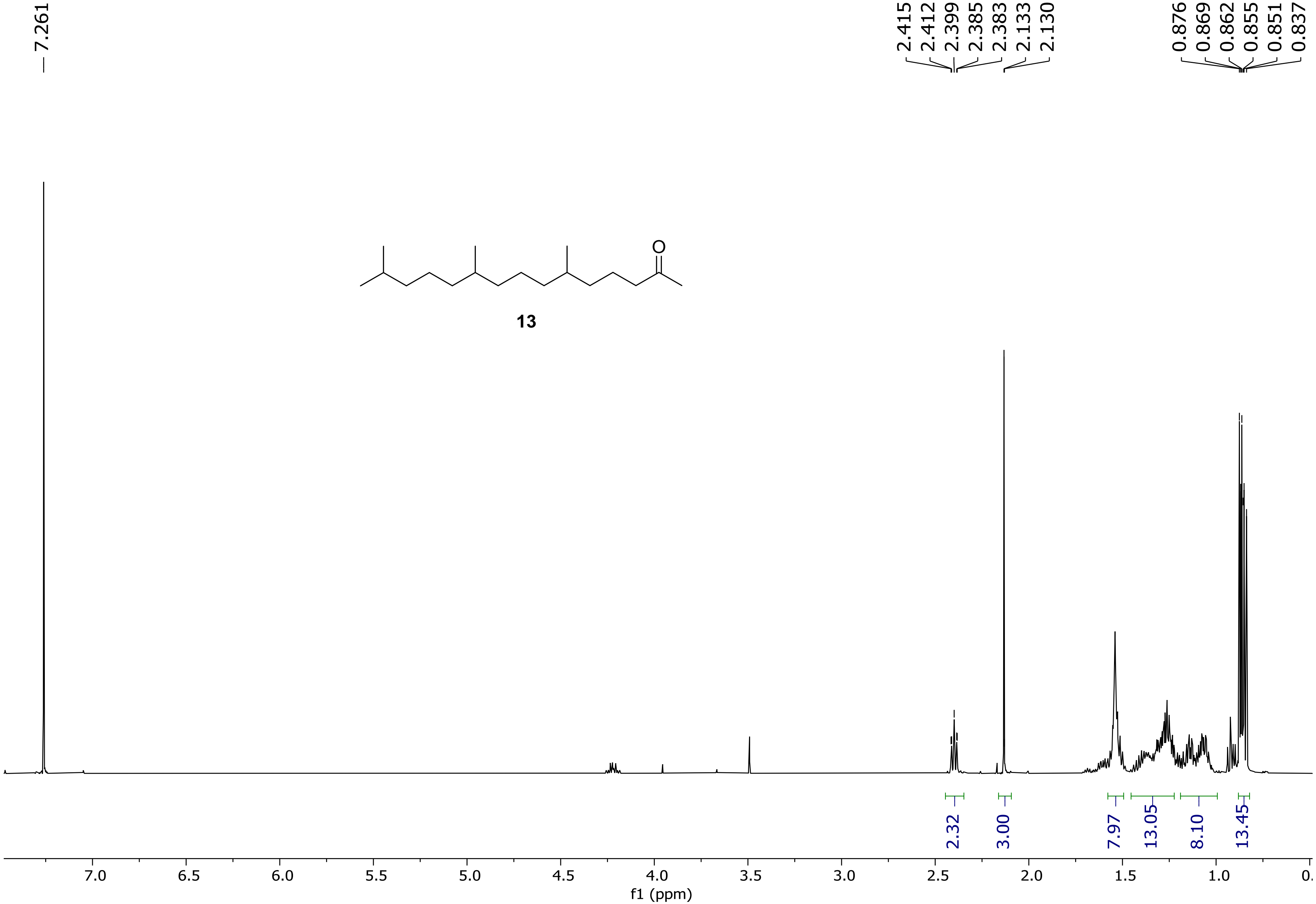

**Figure S26.** $^{1}$H NMR spectrum of **14** in methanol-$d_4$ (700 MHz)

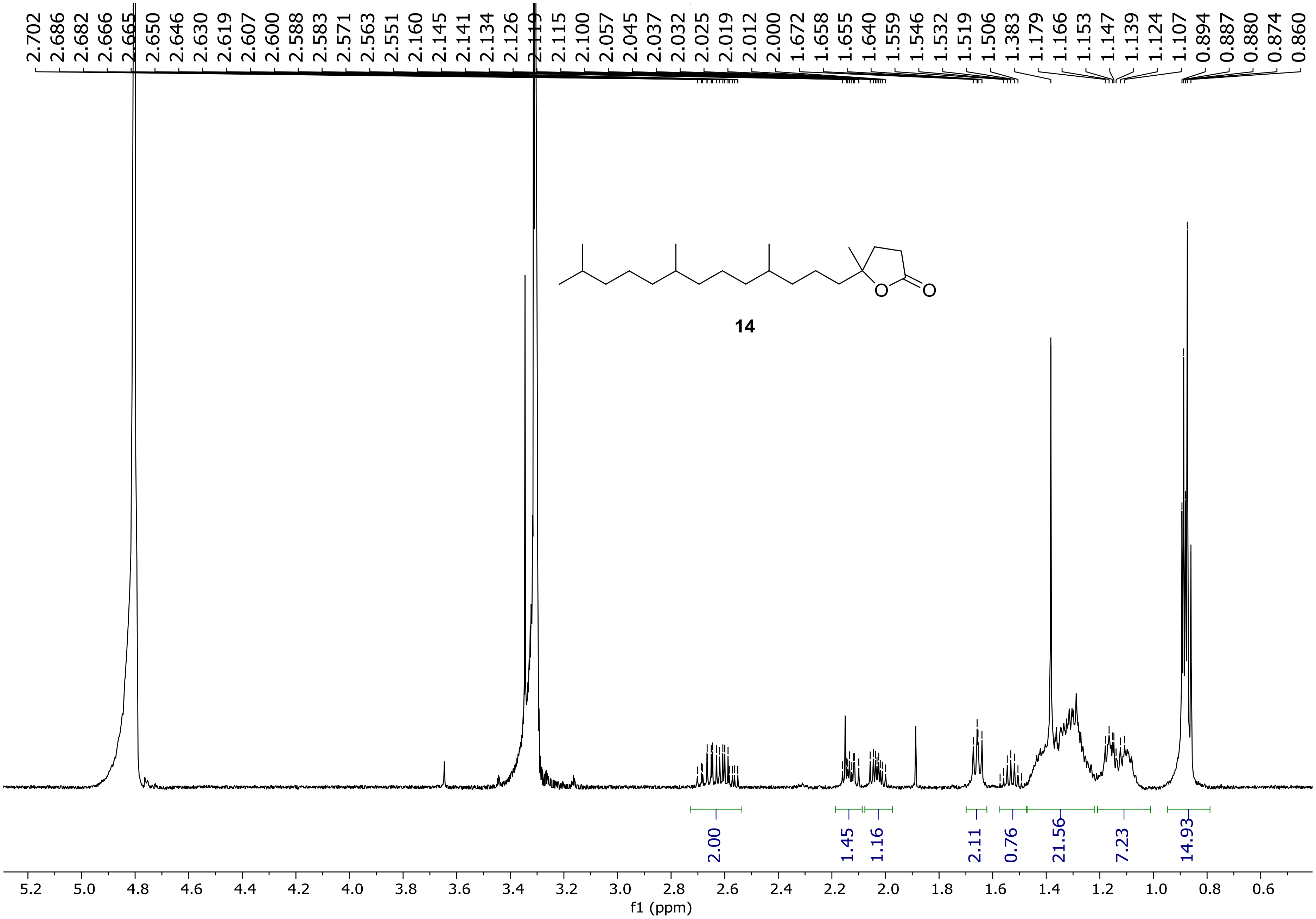

**Figure S27.** $^{13}$C NMR spectrum of **14** in methanol-$d_4$ (175 MHz)

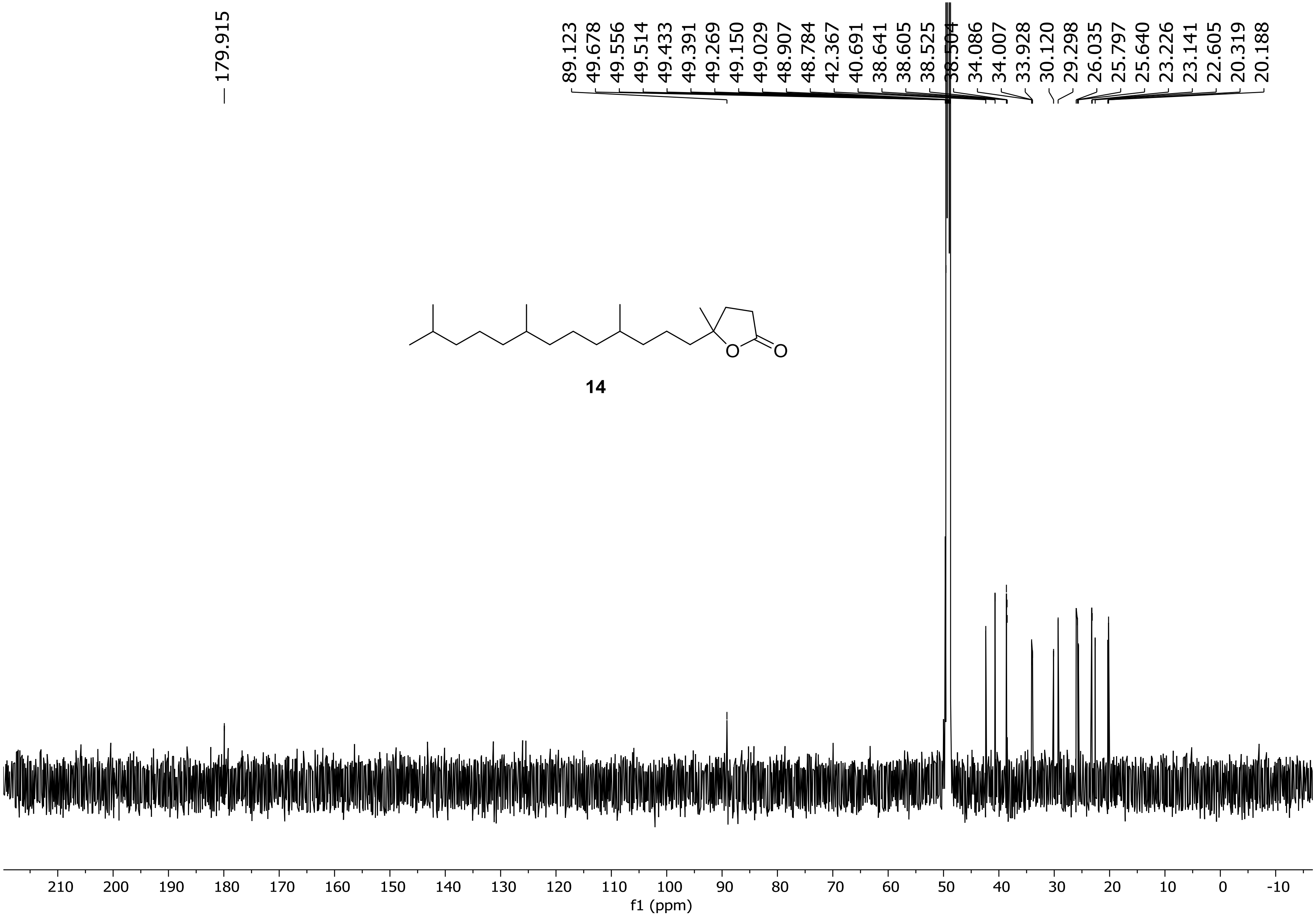

**Figure S28.** $^{1}$H NMR spectrum of **15** in chloroform-*d* (700 MHz)

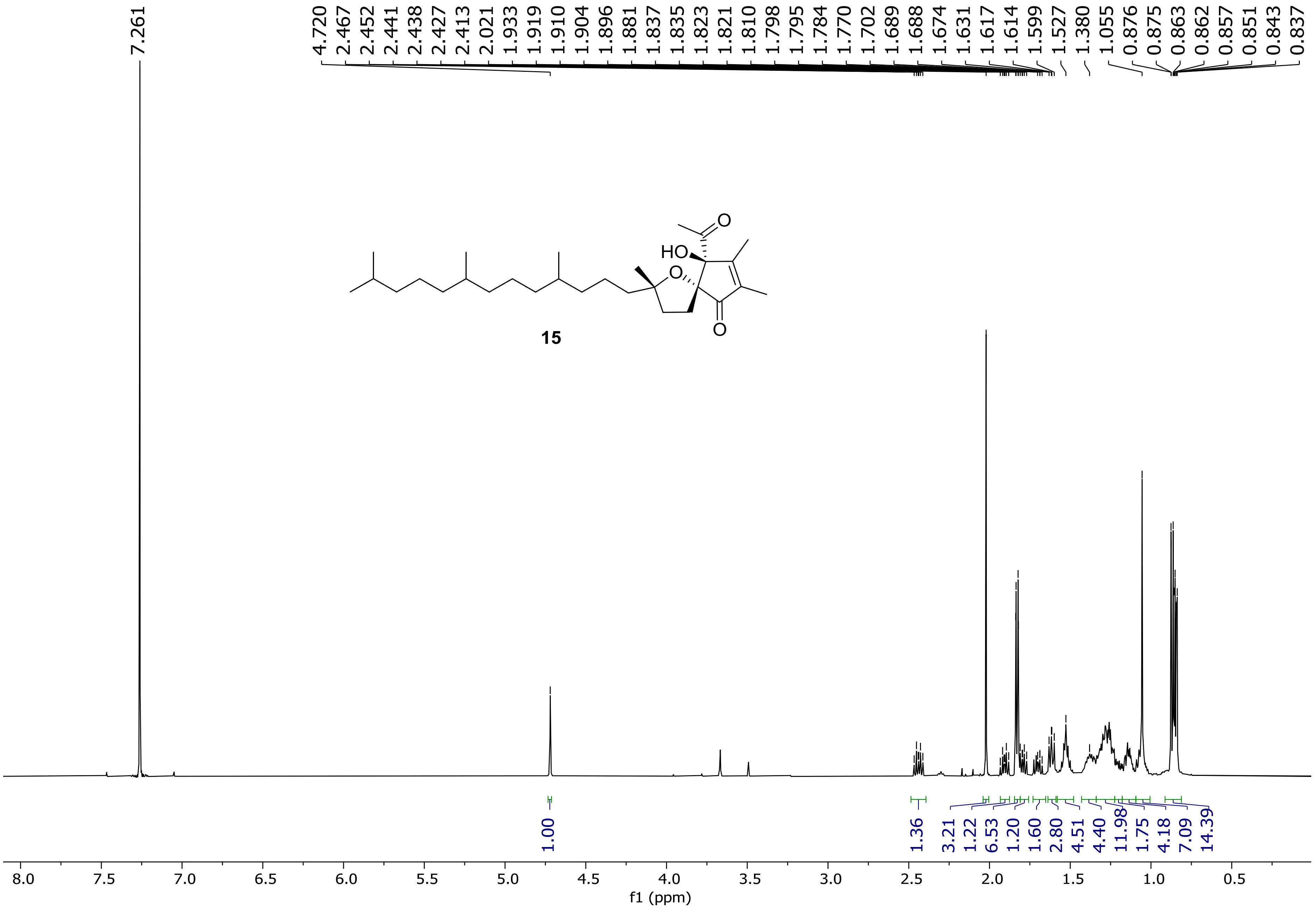

**Figure S29.** $^{13}$C NMR spectrum of **15** in chloroform-*d* (175 MHz)

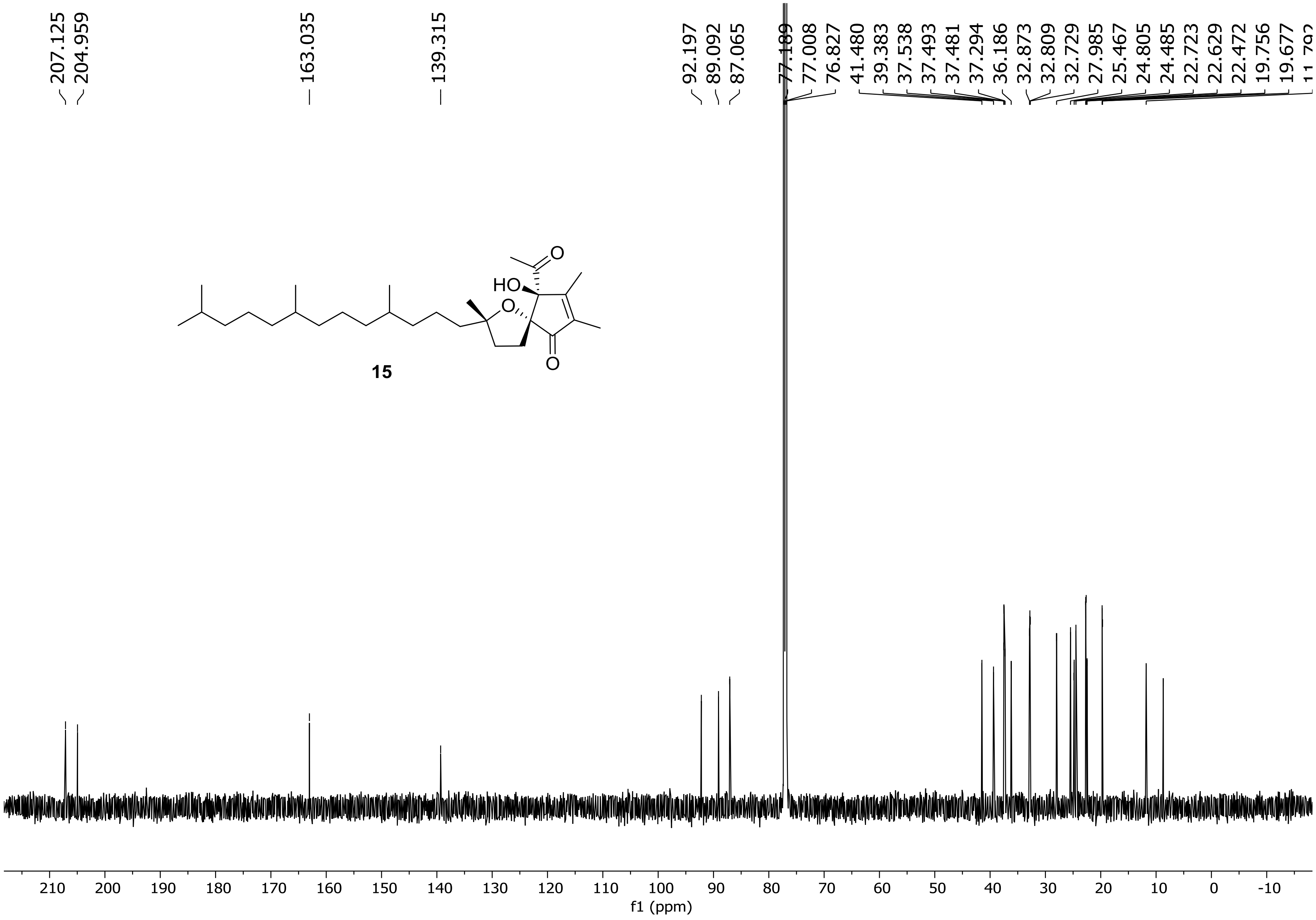

**Figure S30.** $^{1}$H NMR spectrum of **16** in chloroform-*d* (700 MHz)

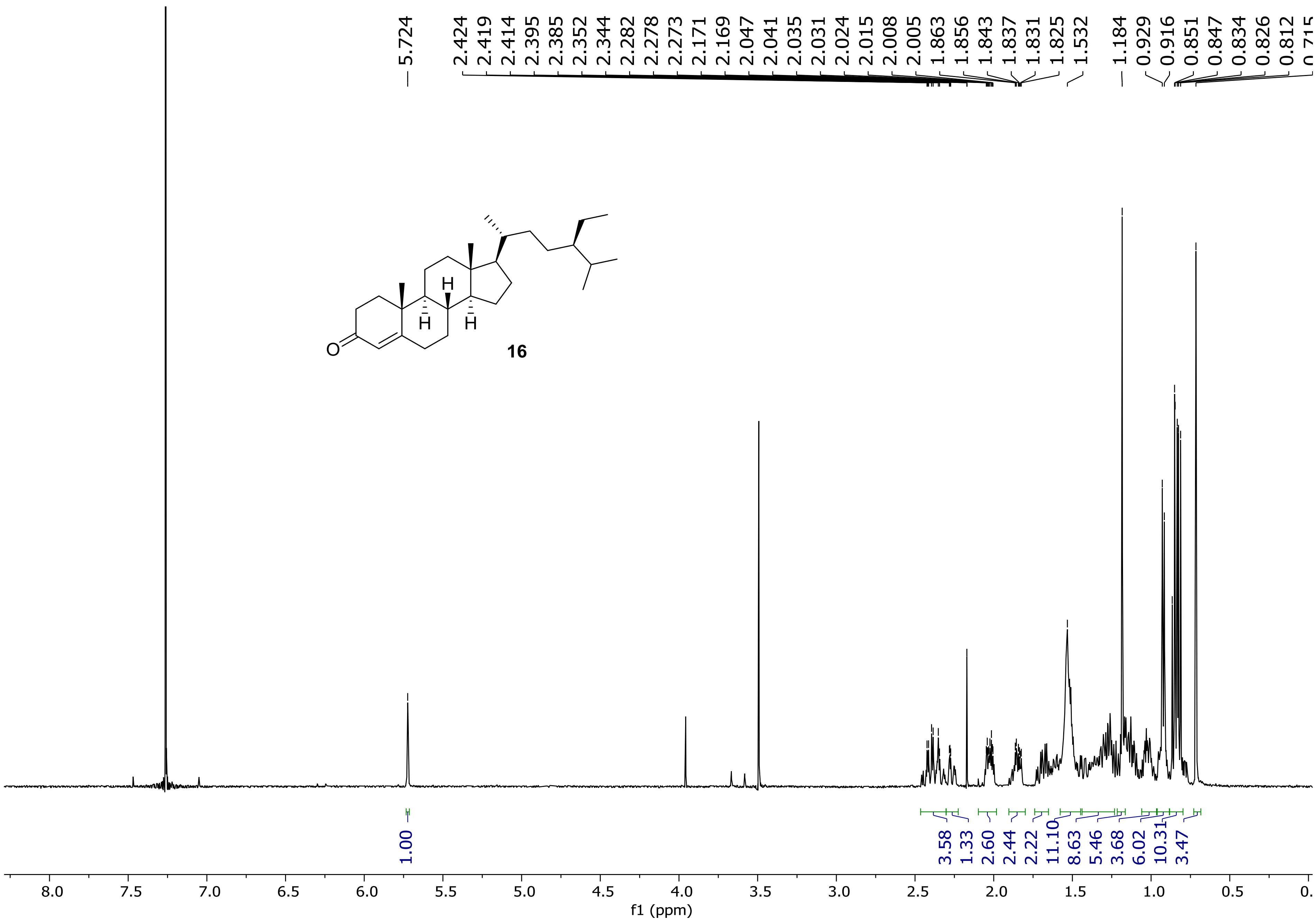

**Figure S31.** $^{13}C$ NMR spectrum of **16** in chloroform-$d$ (175 MHz)

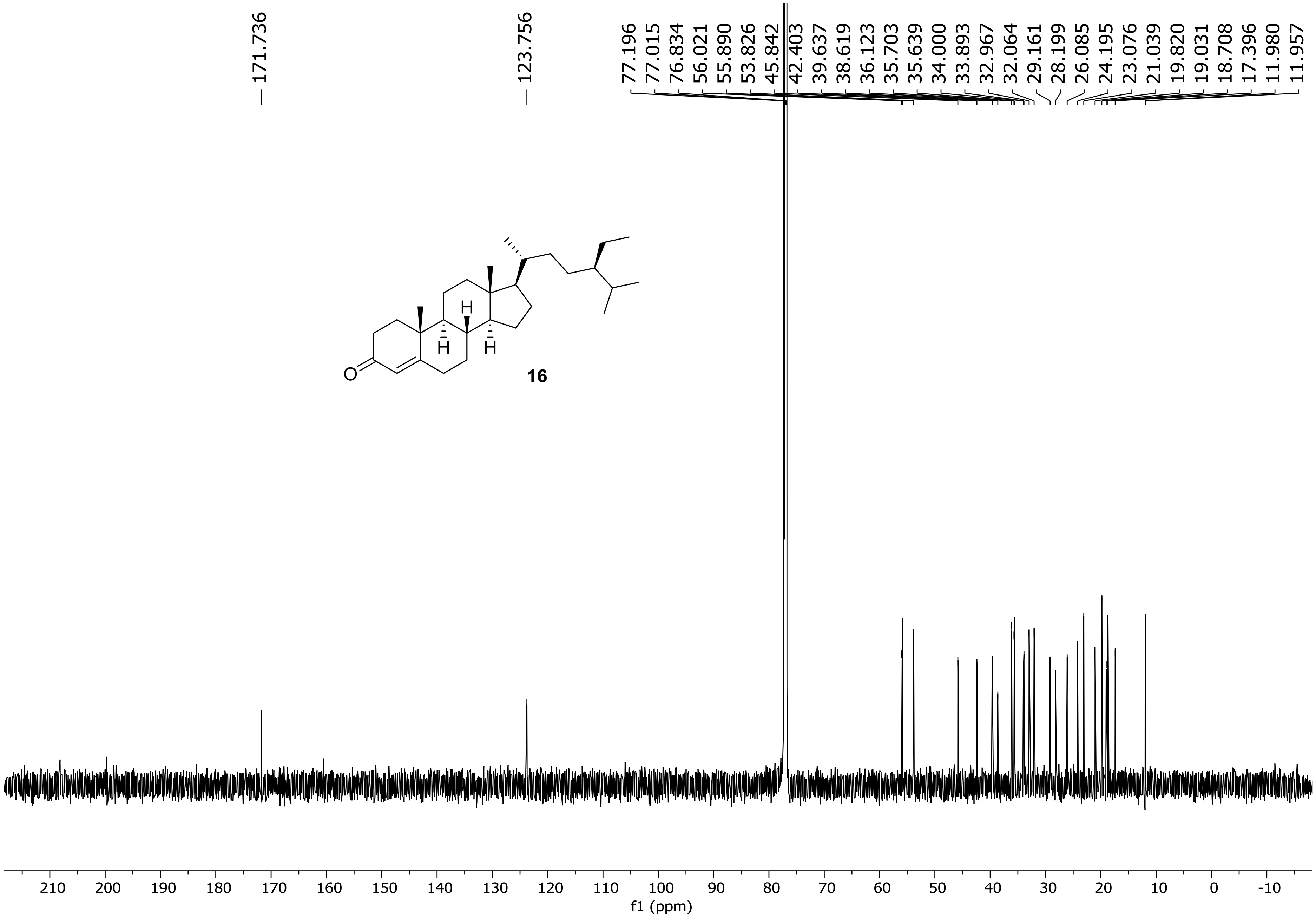

**Figure S32.** $^{1}$H NMR spectrum of **17** in chloroform-*d* (700 MHz)

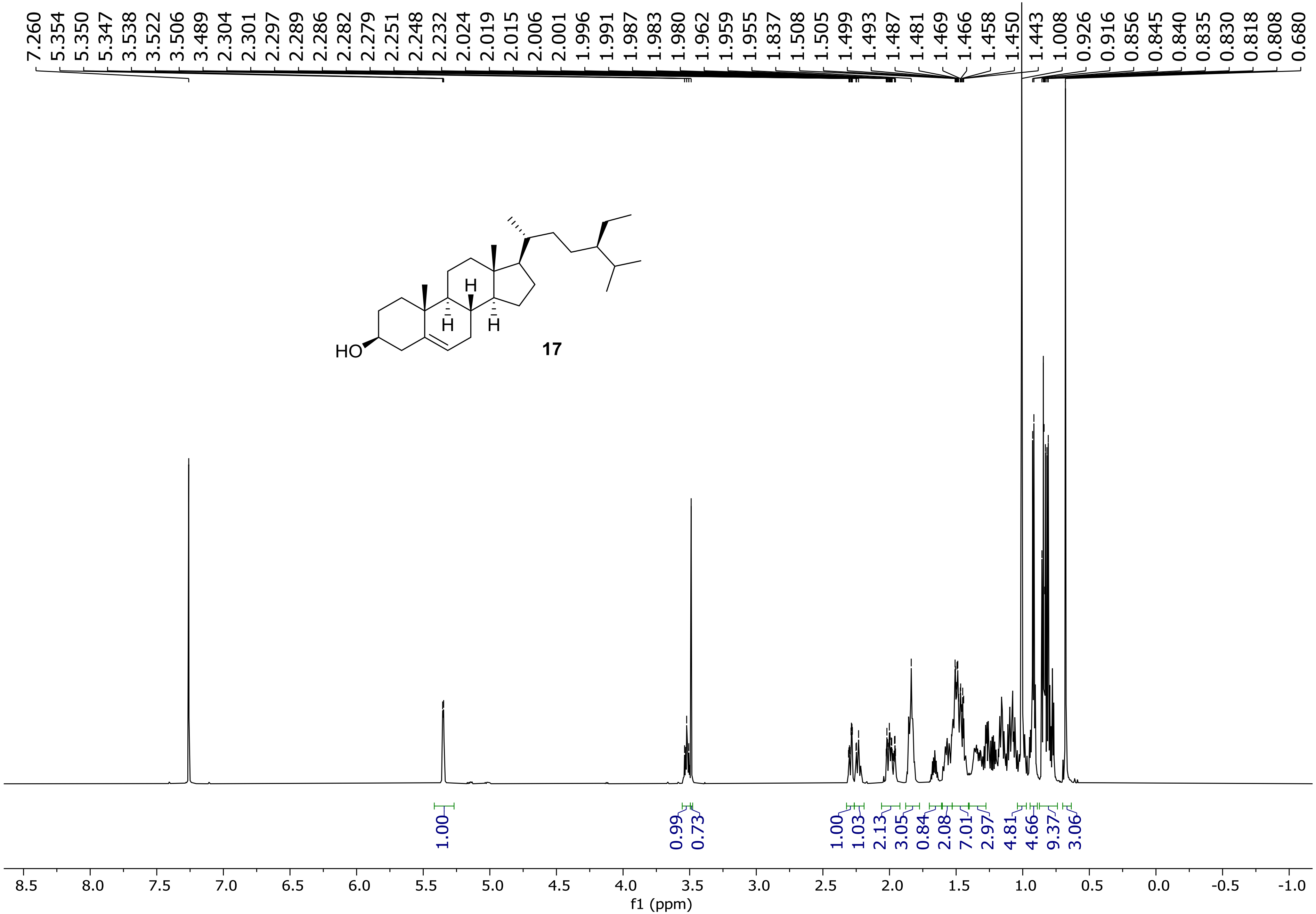

**Figure S33.** $^{13}$C NMR spectrum of **17** in chloroform-*d* (175 MHz)

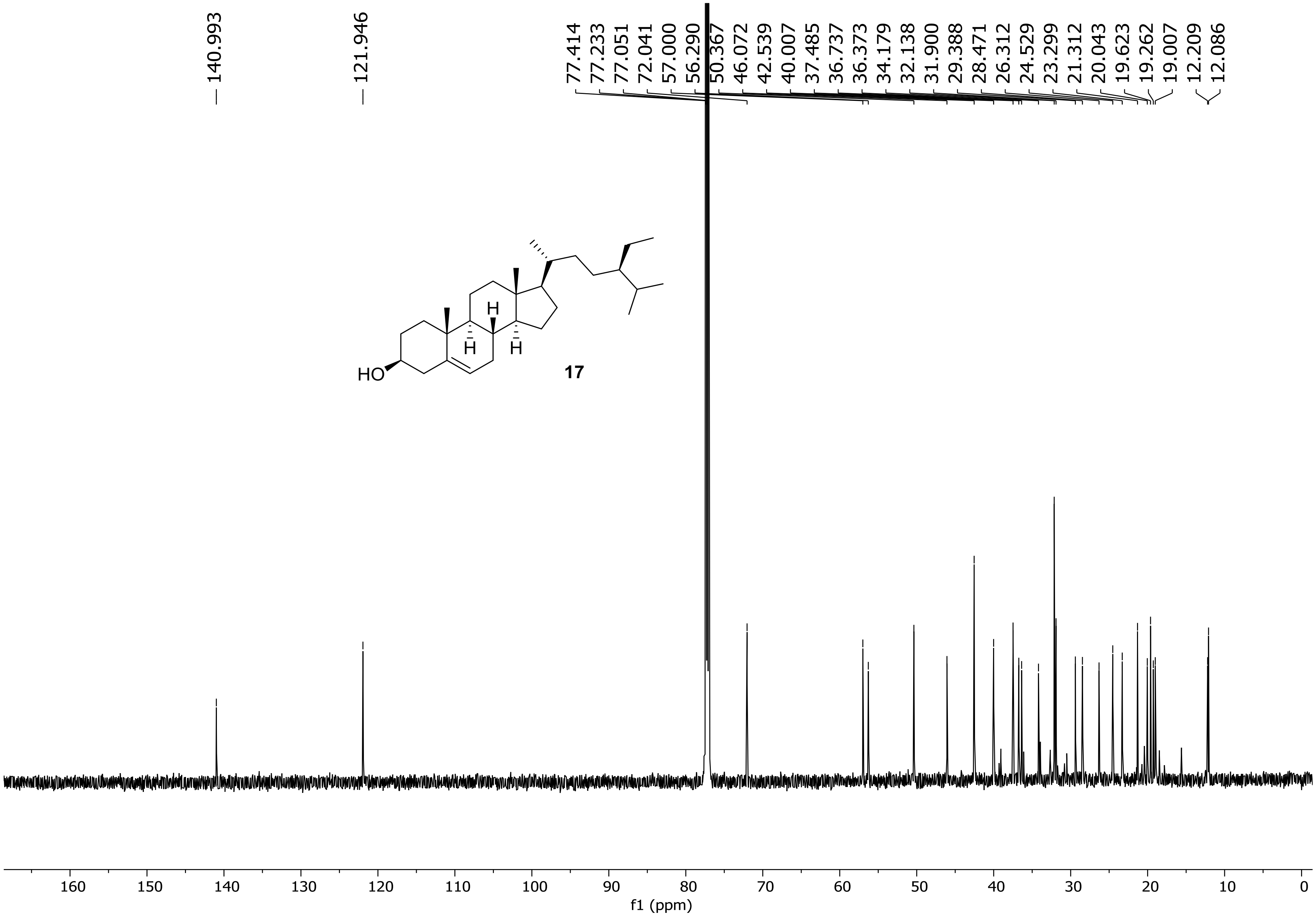

**Figure S34.** $^{1}$H NMR spectrum of **18** in chloroform-*d* (700 MHz)

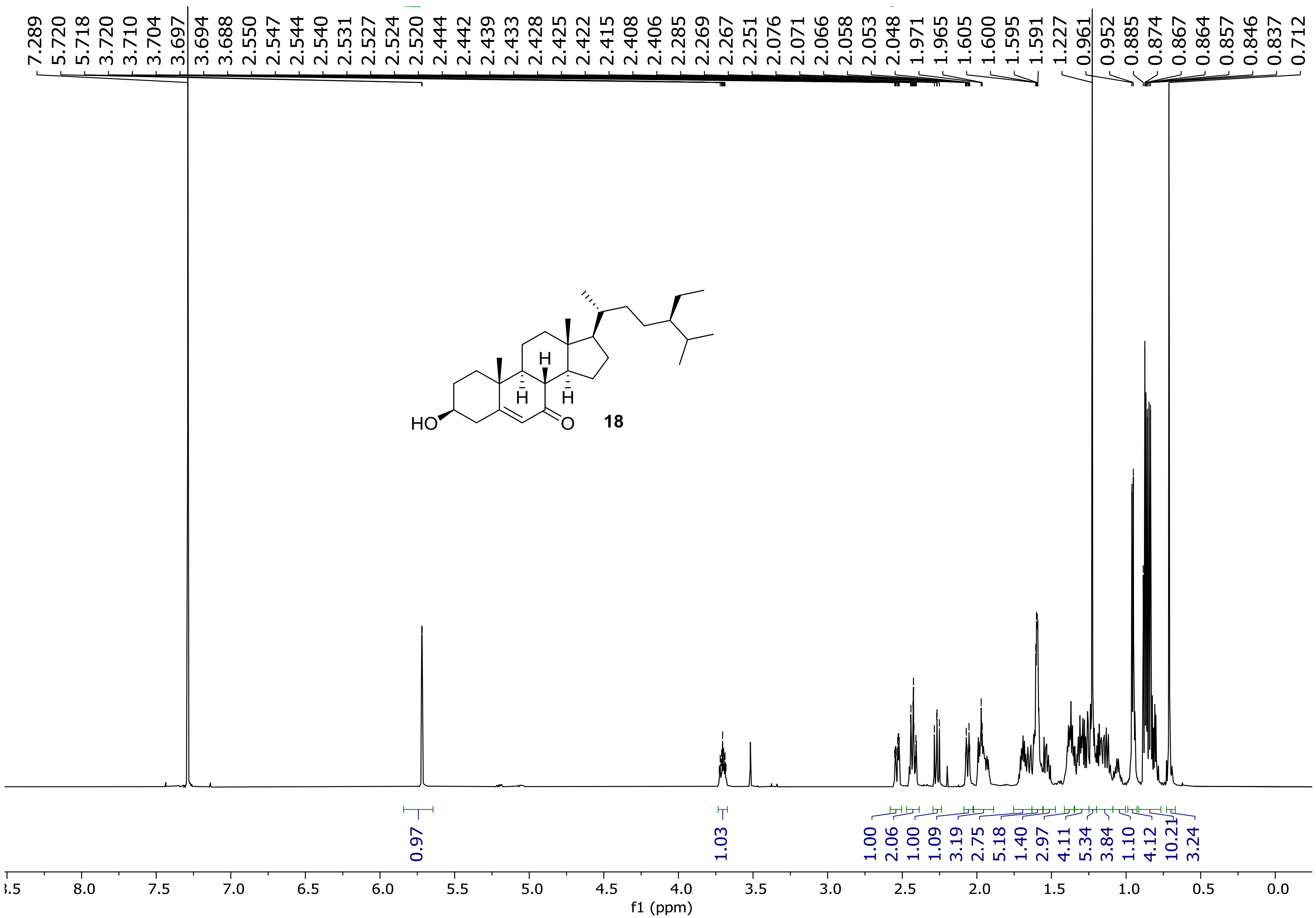

**Figure S35.** $^{13}$C NMR spectrum of **18** in chloroform-*d* (175 MHz)

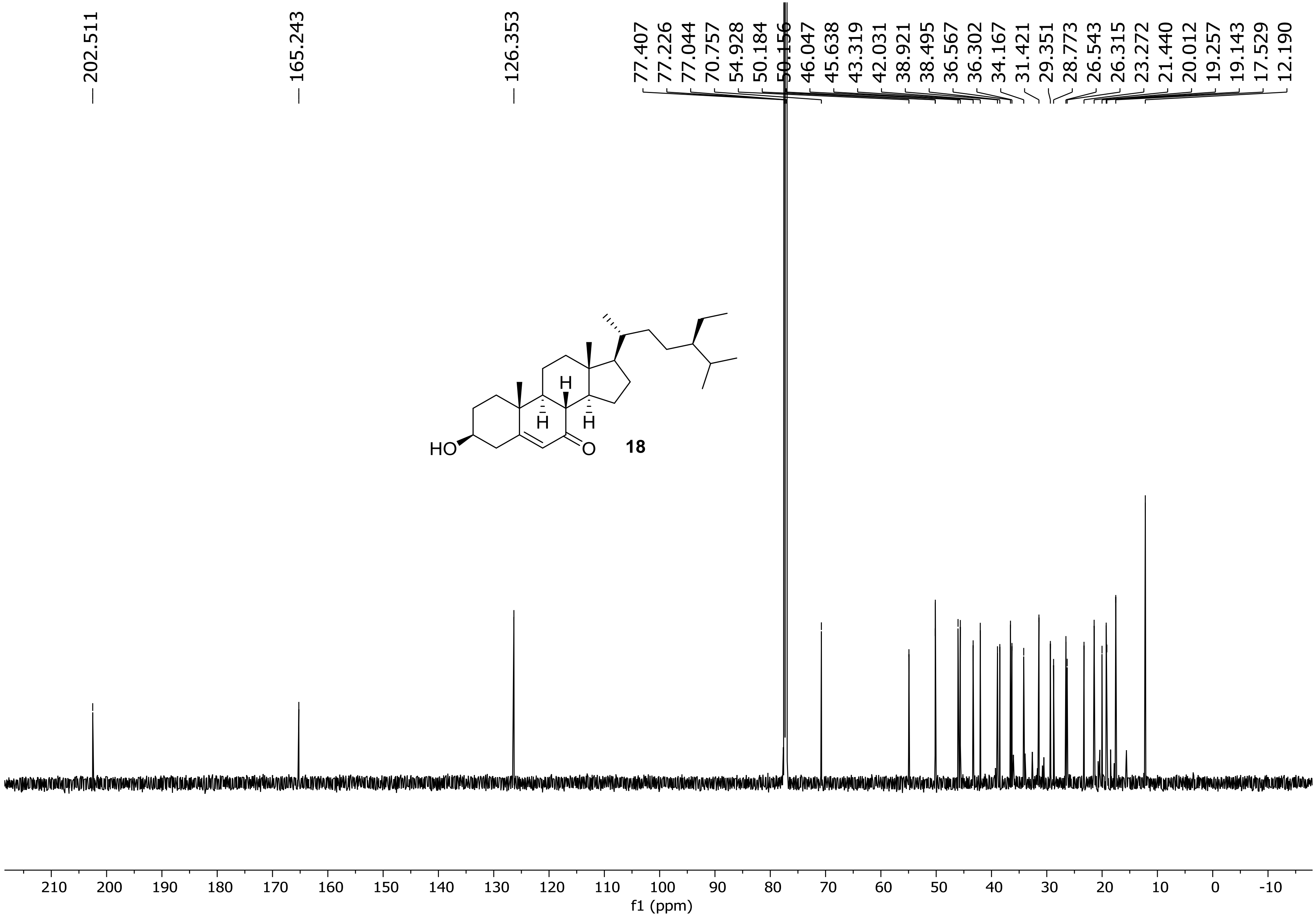

Supplement: Supplementary file 1 [file antioxidants-11-00482-s001.zip › antioxidants-1596794-supplementary.pdf]
